# Natural Diversity in Stomatal Features of Cultivated and Wild *Oryza* Species

**DOI:** 10.1186/s12284-020-00417-0

**Published:** 2020-08-20

**Authors:** Jolly Chatterjee, Vivek Thakur, Robert Nepomuceno, Robert A. Coe, Jacqueline Dionora, Abigail Elmido-Mabilangan, Abraham Darius Llave, Anna Mae Delos Reyes, Apollo Neil Monroy, Irma Canicosa, Anindya Bandyopadhyay, Kshirod K. Jena, Darshan S. Brar, William Paul Quick

**Affiliations:** 1 C4 Rice Center, International Rice Research Institute (IRRI), Los Baños, DAPO BOX 7777, Metro Manila, Philippines; 2 Department of Systems & Computational Biology, School of Life Sciences, University of Hyderabad, Hyderabad, 500046 India; 3 National Institute of Molecular Biology and Biotechnology – University of the Philippines Los Banos, Los Banos, Laguna Philippines; 4 CSIRO Agriculture Flagship, High Resolution Plant Phenomics, GPO Box 1500, Canberra, ACT 2601 Australia; 5 Plant Breeding Division, International Rice Research Institute (IRRI), Los Baños, DAPO BOX 7777, Metro Manila, Philippines; 6 Present Address: School of Agricultural Biotechnology, Punjab Agricultural University, Ludhiana, Punjab India; 7 Department of Animal and Plant Sciences, University of Sheffield, Sheffield, S10 2TN UK

**Keywords:** Stomatal diversity, Wild rice, *Oryza*, Maximum stomatal conductance (anatomical), *g*_max_

## Abstract

**Background:**

Stomata in rice control a number of physiological processes by regulating gas and water exchange between the atmosphere and plant tissues. The impact of the structural diversity of these micropores on its conductance level is an important area to explore before introducing stomatal traits into any breeding program in order to increase photosynthesis and crop yield. Therefore, an intensive measurement of structural components of stomatal complex (SC) of twenty three *Oryza* species spanning the primary, secondary and tertiary gene pools of rice has been conducted.

**Results:**

Extensive diversity was found in stomatal number and size in different *Oryza* species and *Oryza* complexes. Interestingly, the dynamics of stomatal traits in *Oryza* family varies differently within different *Oryza* genetic complexes. Example, the Sativa complex exhibits the greatest diversity in stomatal number, while the Officinalis complex is more diverse for its stomatal size. Combining the structural information with the *Oryza* phylogeny revealed that speciation has tended towards increasing stomatal density rather than stomatal size in rice family. Thus, the most recent species (i.e. the domesticated rice) eventually has developed smaller yet numerous stomata. Along with this, speciation has also resulted in a steady increase in stomatal conductance (anatomical, *g*_max_) in different *Oryza* species. These two results unambiguously prove that increasing stomatal number (which results in stomatal size reduction) has increased the stomatal conductance in rice. Correlations of structural traits with the anatomical conductance, leaf carbon isotope discrimination (∆^13^C) and major leaf morphological and anatomical traits provide strong supports to untangle the ever mysterious dependencies of these traits in rice. The result displayed an expected negative correlation in the number and size of stomata; and positive correlations among the stomatal length, width and area with guard cell length, width on both abaxial and adaxial leaf surfaces. In addition, *g*_max_ is found to be positively correlated with stomatal number and guard cell length. The ∆^13^C values of rice species showed a positive correlation with stomatal number, which suggest an increased water loss with increased stomatal number. Interestingly, in contrast, the ∆^13^C consistently shows a negative relationship with stomatal and guard cell size, which suggests that the water loss is less when the stomata are larger. Therefore, we hypothesize that increasing stomatal size, instead of numbers, is a better approach for breeding programs in order to minimize the water loss through stomata in rice.

**Conclusion:**

Current paper generates useful data on stomatal profile of wild rice that is hitherto unknown for the rice science community. It has been proved here that the speciation has resulted in an increased stomatal number accompanied by size reduction during *Oryza*’s evolutionary course; this has resulted in an increased *g*_max_ but reduced water use efficiency. Although may not be the sole driver of water use efficiency in rice, our data suggests that stomata are a potential target for modifying the currently low water use efficiency in domesticated rice. It is proposed that *Oryza barthii* can be used in traditional breeding programs in enhancing the stomatal size of elite rice cultivars.

## Background

**Fig. 1:**
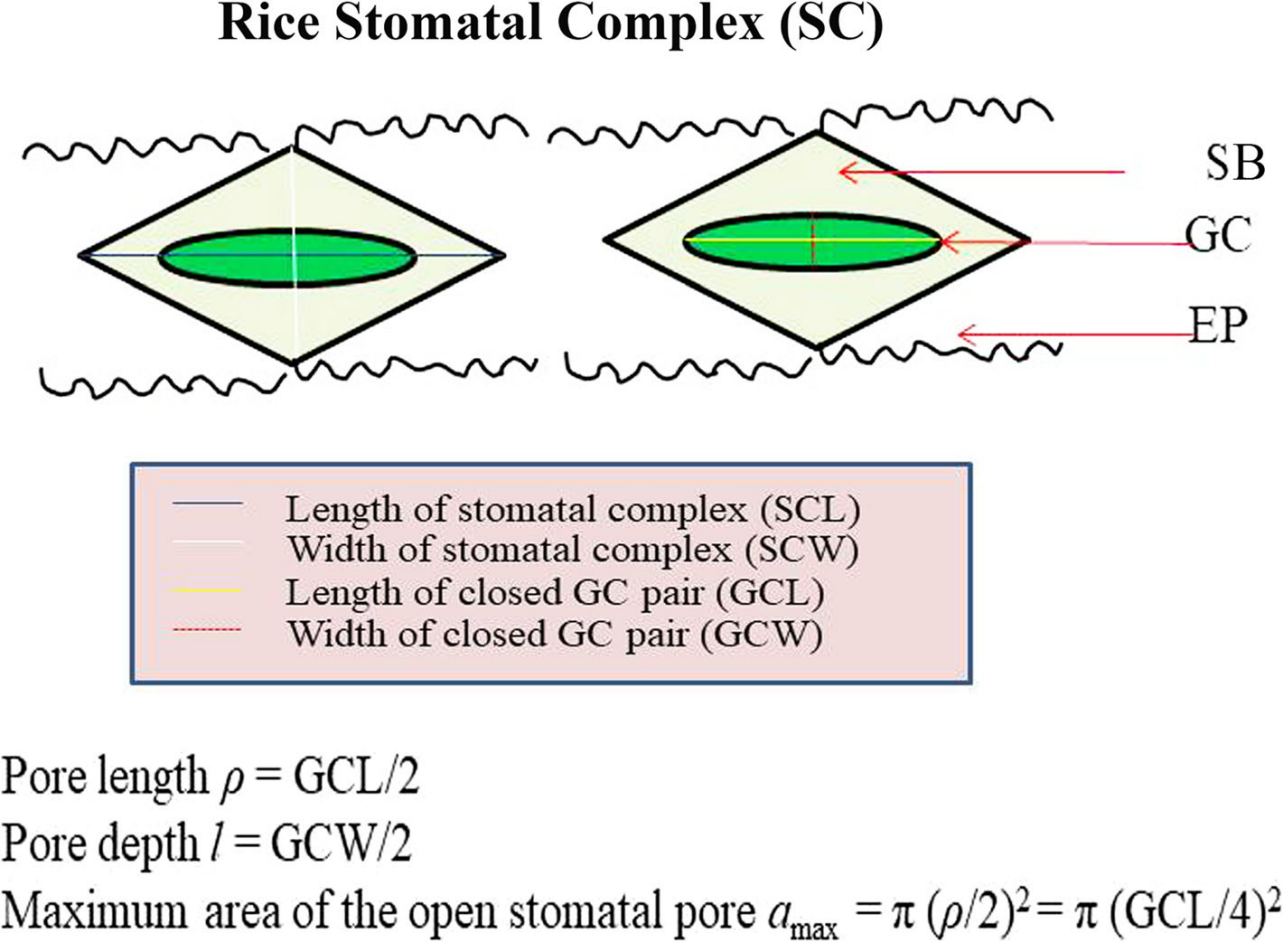
Schematic representation of a pair of rice stomatal complex (SC), accompanied with guard cells (GC), subsidiary calls (SB); and epidermal cells (EP). The diamond shaped SC is structured by two long guard cells, two adjacent subsidiary cells. These are separated by files of epidermal cells. The diagram shows the length (L, the longer diameter of the stomata) and the width (W, the shorter diameter of the stomata) of SC and closed GC pair. GCL and GCW are used to calculate the stomatal pore length (*ρ*) and depth (*l*). *π* is the mathematical constant which is 3.14. Pore length, pore depth and *a*_max_ is calculated according to equation of Franks and Farquhar 2001, 2007. Counting of stomata and physical measurements of all the dimensions and area of SC and GC were performed using Image J image processing software. The long axis of the stoma lies parallel to the leaf lateral axis

Stomata are microscopic pores on the surface of a wide range of plant tissues and most commonly associated with the leaves. Stomatal architecture plays a crucial role in optimizing the gas and water exchange within plant tissues (Cowan 1977; Farquhar et al. 1980; Kanemura et al. 2005; Lawson et al. 2014). Generally, the stomatal complex (SC) in grass species is structured by two guard cells (GC) accompanied by two subsidiary cells (SB) at the sides (Fig. 1, schematic) which work hand in hand. The paired guard cells help in opening of the SC aperture, whereas, the subsidiary or the accessory cells facilitate ionic balance controlling the opening and closing of the GCs (Peterson et al. 2010). Rice stomatal complex (SC) is comprised of two dumbbell-shaped GCs parallel to two SBs at the sides (Luo et al. 2012) separated by a series of long and short epidermal cells (Metcalfe 1960). The density and the size of these pores compared to other epidermal cells controls the maximum conductance of gas and water vapor through these openings. Extensive research has been conducted into the development of stomata in *Arabidopsis* (reviewed in Bergmann and Sack 2007), involving the function of *Epidermal Patterning Factor* (*EPF*) gene cascades (Hara et al. 2007), and molecular regulators of opening and closing of the guard cells (Misra et al. 2015; Murata et al. 2015), which are believed to work globally across a range of species including rice. But, surprisingly, the diversity of these tiny organs has not yet been fully documented in rice and rice wild species. Being an important regulator of many of the physiological processes including, photosynthesis, transpiration, respiration etc., therefore, it is crucial to understand the diversity in the architecture of these pores to determine if any of the stomatal traits can be incorporated into rice breeding program for improving yield of this crop (Jones 1987).

Rice family consists of twenty four *Oryza* species, of which two are domesticated (*O. sativa* and *O. glaberrima*) and others are commonly known as wild rice or non-domesticated rice (Vaughan 1994). Considering the reproductive barrier and distance in genetic relatedness, this large *Oryza* family is grouped into four different “*Oryza* complexes”, which include eleven different rice genetic backgrounds in total, typically named from AA to KKLL (Tateoka 1962; Vaughan 1994). The Sativa complex contains eight diploid *Oryza* species, the Officinalis complex contains nine both diploid and tetraploid species, and the Meyeriana and Ridleyi complexes contain four tetraploid species. The two other *Oryza* species, *O. brachyantha* and *O. coarctata* are not included in any of these complexes, but genetically belong to the *Oryza* family (Lu et al. 2009). Collectively the wild species possesses a unique collection of morphological, anatomical (Chatterjee et al. 2016; Giuliani et al. 2013) and physiological traits (Zhao et al. 2010; Kondamudi et al. 2016) that are still unused for improvement of cultivated rice. In our previous study we have characterized these wild rice species for leaf characters, and documented significant diversity in leaf morphological and anatomical characters, and studied the evolution of leaf traits in rice family (Chatterjee et al. 2016). These diverse leaves are expected to hold a fair amount of diversity in the leaf epidermal features like stomata as well, which is examined thoroughly in this study.

In our present study we have characterized the stomatal character of a total of twenty three *Oryza* species (Table 1) (except the wild species *O. schlechteri*). We have studied their number of stomata present per unit leaf space; length, width, area of the individual stomatal complex; length and width of closed guard cell pair. The maximum anatomical conductance of stomata (*g*_max_) according to their structures was calculated using Farquhar model (Franks and Farquhar 2001). These traits are crucial for plants growth and water balance in plants and widely used in crops (Shahinnia et al. 2016). The (*g*_max_) is the theoretical maximum rate of water vapor exchange through stomata that can be possible providing optimum growth condition and it depends on stomatal morphology. It is higher than the actual operational conductance (*g*_op_) that happens in field. Anatomical *g*_max_ tallies well with plants diffusive *g*_max_ when measured in laboratory condition (Dow et al. 2014a, 2014b). We have also measured the dry matter carbon isotope discrimination Δ^13^C values for these species. The data was used to understand the correlations among stomatal traits, along with other leaf traits like, leaf length, width, vein numbers, height, width etc. The leaf traits were previously described in detail in Chatterjee et al. 2016. The present study documents substantial diversity in stomatal structure in the *Oryza* family, which have been inter-related with its functional features to decode how the physiological parameters altered by the changes in stomatal structure in rice.

**Table 1:** *Oryza species,* complexes, and IRGC accession numbers. According to standard wild rice classification and recent *Oryza* phylogenetic research *Oryza* species of rice family are classified into four different complexes (Sativa, Officinalis, Meyeriana, and Ridleyi) that contain different *Oryza* genome types (Tateoka 1962; Vaughan 1994). *Oryza brachyantha* and *O. coarctata* stand alone because of their genetic distance from the cultivated rice, but are still included in the wild rice family (Lu et al. 2009)

Classification		Genome	*Oryza* species	IRGC Accession number
Sativa complex	Asian species	AA	*O. sativa*	IR 64–21
		AA	*O. rufipogon*	106424
		AA	*O. nivara*	80723
		AA	*O. glumaepatula*	106242
	African species	AA	*O. glaberrima*	103544
		AA	*O. barthii*	106017
		AA	*O. longistaminata*	110404
		AA	*O. meridionalis*	105301
Officinalis complex		BB	*O. punctata*	105690
		CC	*O. eichingeri*	101422
		BBCC	*O. minuta*	101141
		CC	*O. officinalis*	100896
		CC	*O. rhizomatis*	105659
		CCDD	*O. alta*	105143
		CCDD	*O grandiglumis*	106241
		CCDD	*O. latifolia*	105173
		EE	*O. australiensis*	100882
Meyeriana complex		GG	*O meyeriana*	89241
		GG	*O. granulata*	102118
Ridleyi complex		HHJJ	*O. ridleyi*	100821
		HHJJ	*O longiglumis*	105148
Others		FF	*O. brachyantha*	101232
		KKLL	*O. coarctata*	104502

## Results

A schematic diagram of a pair of stomatal complex (SC) is illustrated in Fig. 1. Table 2 shows the parameters we have measured to quantify the number, structural and functional features of stomata (SC) and guard cells (GC) on both abaxial and adaxial leaf surfaces. Variations in these traits are described in detail in the following sections. The traits are mainly compared to that of a popular high yielding rice cultivar IR64 (*Oryza sativa*) that belongs to the Sativa complex.

**Table 2:** List of traits studies for stomatal structure and functional variation

Type	Abbreviation	Unit	Explanation
Structural traits	SD_ab_	count	Stomatal density / mm^2^ at the abaxial side of leaf
	SD_ad_	count	Stomatal density / mm^2^ at the adaxial side of leaf
	SCL_ab_	μm	Length of the stomatal complex at the abaxial side of leaf
	SCL_ad_	μm	Length of the stomatal complex at the adaxial side of leaf
	SCW_ab_	μm	Width of the stomatal complex at the abaxial side of leaf
	SCW_ad_	μm	Width of the stomatal complex at the adaxial side of leaf
	SCA_ab_	μm^2^	Area of the stomatal complex at the abaxial side of leaf
	SCA_ad_	μm^2^	Area of the stomatal complex at the adaxial side of leaf
	GCL_ab_	μm	Length of the closed guard cell pair at the abaxial side of leaf
	GCL_ad_	μm	Length of the closed guard cell pair at the adaxial side of leaf
	GCW_ab_	μm	Width of closed guard cell pair at the abaxial side of leaf
	GCW_ad_	μm	Width of closed guard cell pair at the adaxial side of leaf
	SC_distance_ab_	μm	Inter-stomatal distance along the leaf length at the abaxial side of the leaf
	SC_distance_ad_	μm	Inter-stomatal distance along the leaf length at the adaxial side of the leaf
	EPL_ab_	μm	Length of the epidermal cell at the abaxial side of leaf
	EPW_ab_	μm	Width of the epidermal cell at the abaxial side of leaf
	VD	count	Vein density mm^−1^ leaf lateral space
	VH	μm	Height of minor vein
	VW	μm	Width of minor vein
	LL	cm	Leaf length from base of the leaf to tip
	LW	cm	Leaf width measured from margin to margin at the middle of the leaf
	LA_total_	cm^2^	Approximate leaf area (LL x LW)
	LT	μm	Leaf thickness measured as the height of the leaf transverse section image at the middle of leaf
	Total stomata__ab_	count	Total number of stomata in the abaxial side computed as SD_ab_ x LL x LW
Functional traits	g_max_ab_	mol m^2^ s^1^	Stomatal conductance to water vapour at the abaxial side of leaf
	g_max_ad_	mol m^2^ s^1^	Stomatal conductance to water vapour at the adaxial side of leaf
	g_max_total_	mol m^2^ s^1^	Total stomatal conductance to water vapour
	g_max_ad:ab_	ratio	Ratio between adaxial vs abaxial stomatal conductance
	∆^13^C	‰	C^13^ vs. C^12^ discrimination ratio in leaf dry matter

Leaf water conductances are derived from the stomatal structural features according to Franks and Farquhar 2001. Leaf traits are measured in our previous study by Chatterjee et al. 2016

### Diversity in Stomatal Complex

**Fig. 2:**
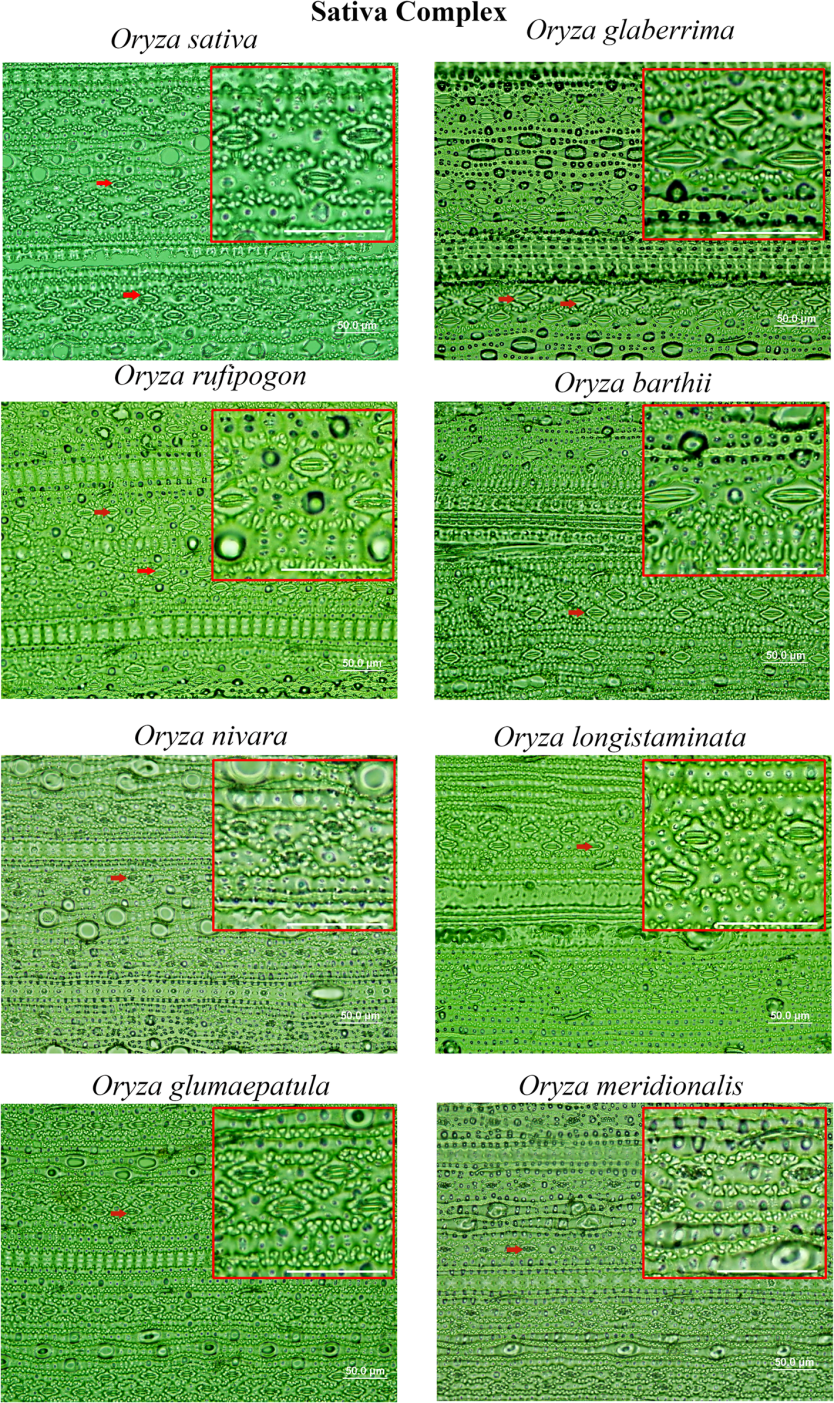
Stomatal diversity in *Oryza* “Sativa Complex”. Files of stomata are arranged in parallel on the surface along the length of the leaf. Some of the stomatal complexes are shown by red arrows. Scale bar is 50.0 μm. The images are captured from the abaxial epidermal layers after scraping of leaf tissues, under 40x magnification with 10x eyepiece of a BX51 light microscope (Olympus). The oval shapes are the marks of trichomes on the leaf. Difference in number and size in stomata is very prominent in the species. Note the larger stomata of *O. glaberrima*, *O. barthii* and smaller stomata of *O. nivara* and *O. meridionalis* as compared to *Oryza sativa*. Red lined boxes are the zoomed part of the leaves showing stomatal features

**Fig. 3:**
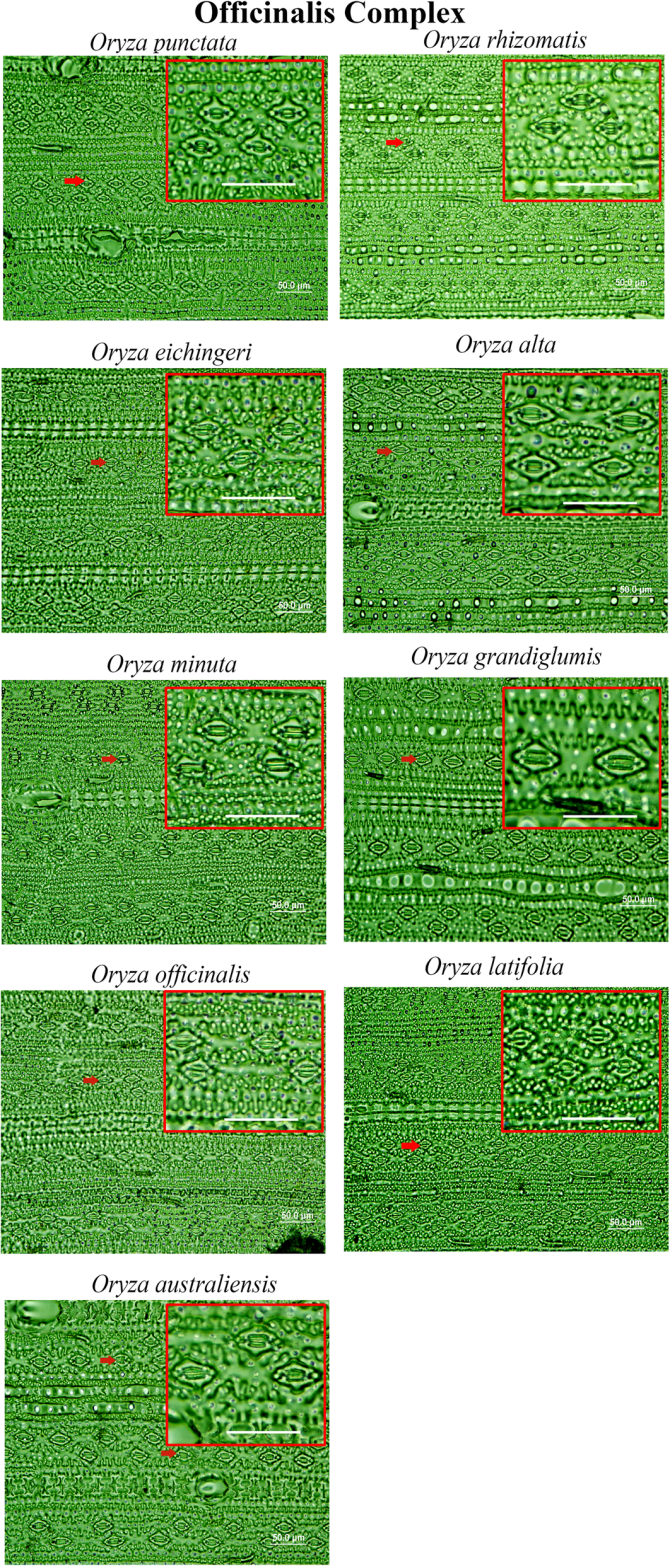
Stomatal diversity in *Oryza* “Officinalis Complex”. The images are captured from the abaxial epidermal layers after scraping of leaf tissues, captured under 40x magnification with 10x eyepiece using BX51 (Olympus). The oval shapes are the marks of trichomes on the leaf. The Officinalis complex varies more in terms of the size of stomata than the number of stomata. Some of the stomata are marked by red arrows. Scale bar is 50.00 μm. Red lined boxes are the zoomed part of the leaves showing stomatal features

**Fig. 4:**
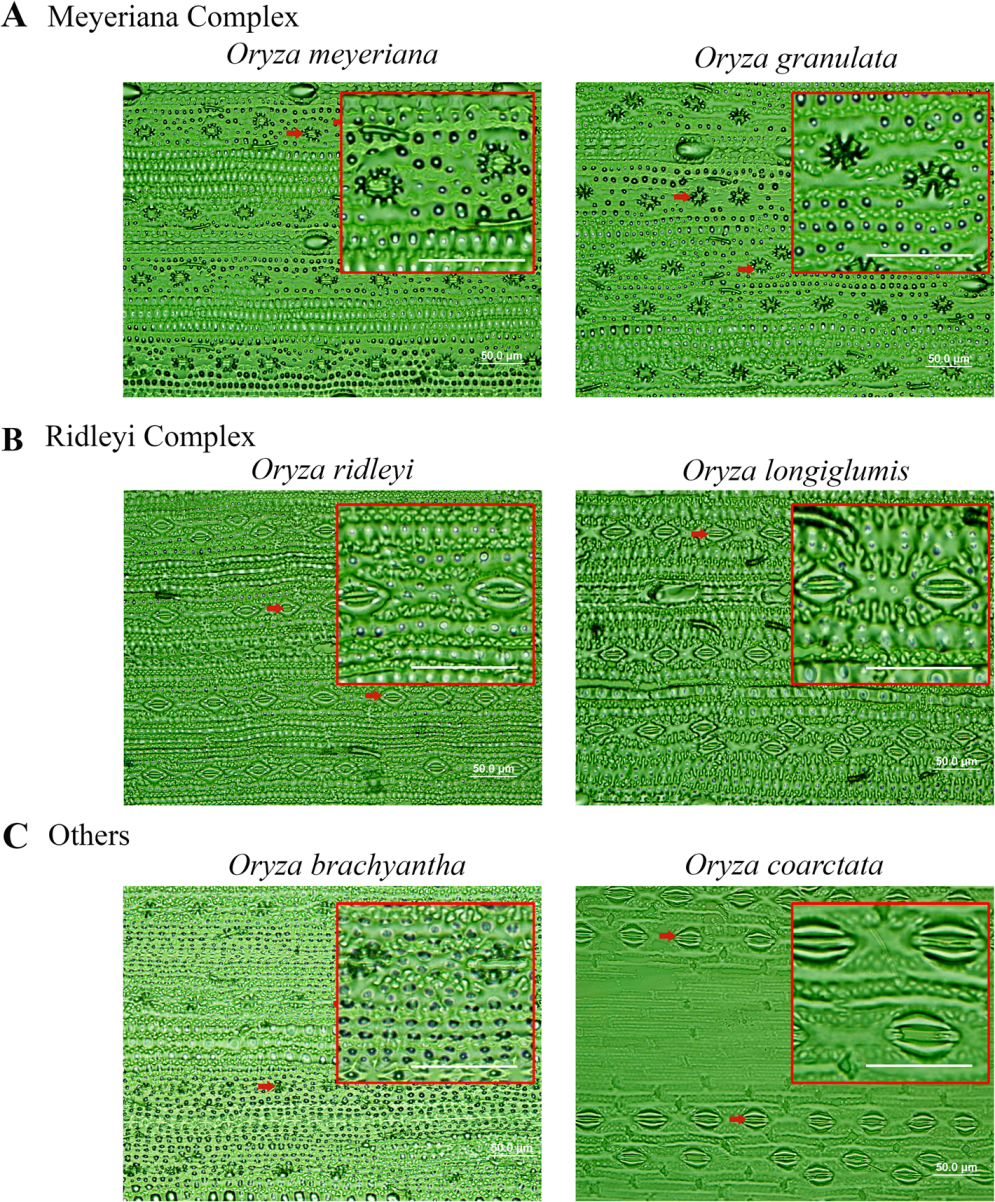
Stomatal diversity in M-R-O, i.e., Meyeriana (**a**), Ridleyi (**b**) and Other (**c**) complexes. Stomata are much smaller in *O. meyeriana*, *O. granulata* and *O. brachyantha*; and significantly larger in *O. ridleyi*, *O. longiglumis* and *O. coarctata* as compared to *O. sativa* (Fig. 2). Some of the stomata are marked by red arrows. Scale bar is 50.00 μm. Red lined boxes are the zoomed part of the leaves showing stomatal features

**Fig. 5:**
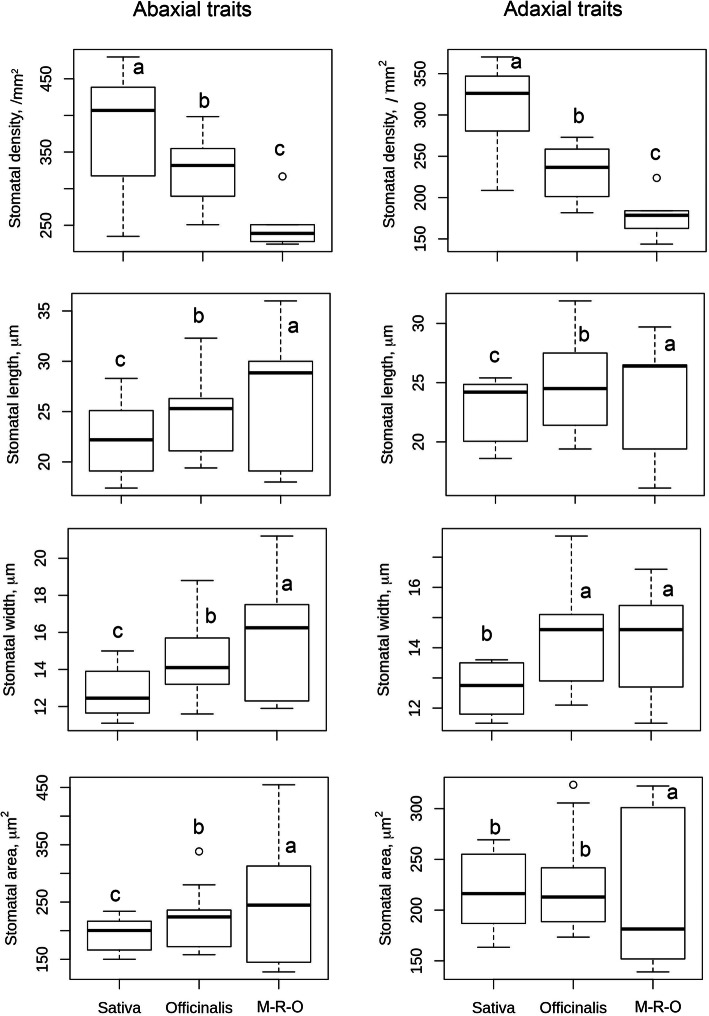
Box plots show number and structural diversity of stomatal complex in *Oryza*; Sativa, Officinalis and M-R-O (Meyeriana, Ridleyi and Others) complexes. A detailed value for each species, and range of diversity for each complex are given in Table S1 and in Table S2. Boxes shows 25th to 75th percentile, horizontal line represents the median and whiskers indicate the minimum and maximum values. Different letters represent significant difference in different complexes for the concerned trait (at *P* < 0.05). The values on the adaxial leaf side are of excluding *O. coarctata*. Sativa complex contains eight, Officinalis complex contains nine, and Meyeriana, Ridleyi and Others (M-R-O) together contains six *Oryza* species. Stomatal number is counted from 15 random images taken from the middle portion of the leaf from 3 leaves per species. SCL, SCW, SCA are measured from 25 random stomatal images for each species

Stomatal complexes are found to vary both in terms of number and size in *Oryza* species (Fig. 2, 3, 4). Significant variations are found at the species level, (Table S1) and at different *Oryza* complex levels (Fig. 5, Table S2). These are explained in detail in the following sections.

#### Stomatal Density (SD)

A detailed SD scoring for each species (Table S1) shows that the trait varies from 224.3 mm^− 2^ (*O. longiglumis,* Ridleyi complex) to 479.8 mm^− 2^ (*O. nivara,* Sativa complex) in the abaxial side, and from 143.7 mm^− 2^ (*O. meyeriana,* Meyeriana complex) to 362.7 mm^− 2^ (*O. rufipogon,* Sativa complex) on the adaxial side. Among the cultivated species, SD is higher in African cultivated rice *O. glaberrima* (SD_ab_ = 436.5 mm^− 2^, SD_ad_ = 328.6 mm^− 2^) compared to Asian cultivated rice *O. sativa* (SD_ab_ = 393.7 mm^− 2^, SD_ad_ = 317.5 mm^− 2^). Except in *O. australiensis,* the SD is always higher on the abaxial leaf side. For other species, the difference in SD_ab_ and SD_ad_ varies from a number of 3.2 mm^− 2^ (in *O. longistaminata*) to 173 mm^− 2^ (in *O. officinalis*). This eventually creates an impact in the ratio of the adaxial vs. abaxial stomatal number, which varies from 0.51 (*O. officinalis*) to 1.05 (in *O. australiensis*).

SD varies significantly among different *Oryza* complexes too. The average SD is highest in Sativa complex (Fig. 5). The species of the Sativa complex have the highest stomatal density mm^− 2^ leaf area, ranging from 234.9 mm^− 2^ to 479.8 mm^− 2^ on the abaxial and from 208.7–370.2 mm^− 2^ on the adaxial side, and thus possess the highest diversity for this trait (SD_ab_ = 64.42%, SD_ad_ = 51.96%, Table S2). SD varies from 250.8 to 398.4 mm^− 2^ on the abaxial and from 181.7 to 273.0 mm^− 2^ on the adaxial side in the Officinalis complex. The SD diversity in the species of Meyeriana, Ridleyi and Other (M-R-O) complexes individually are quite low compared to the Sativa and the Officinalis complex, and the actual number collectively ranges from 224.3 to 316.7 mm^− 2^, and from 143.7 to 223.8 mm^− 2^ of leaf area in the abaxial and adaxial leaf surfaces respectively. The actual number of stomata is minimal in the Ridleyi complex. *Oryza rufipogon*, *O. nivara*, *O. glaberrima* and *O. meridionalis* have significantly higher stomatal numbers (range SD_ab_ = 420–479.8 mm^− 2^, SD_ad_ = 331.4–370.2 mm^− 2^) on both abaxial and adaxial surfaces compared to IR64.

#### Diversity in Stomatal Length (SCL), Width (SCW) and Area (SCA)

Both the abaxial and adaxial SCL varies significantly in different *Oryza* complexes (Fig. 5), SCL measurements show longer stomata on average in Ridleyi and Other complexes, compared to that in Sativa and Officinalis and Meyeriana (Table S2). Overall, the SCL varies almost two folds among the *Oryza* species, from 17.4 μm (in *O. meridionalis,* Sativa complex) to 36.0 μm (in *O. coarctata,* undefined complex) on the abaxial side and from 16.1 μm (*O. granulata,* Meyeriana complex) to 31.9 μm (*O. grandiglumis,* Officinalis complex) on the adaxial side (Table S1). This trait is most diverse in the species of Officinalis complex, Table S2, (GD, SCL_ab_ = 51.7%, SCL_ad_ = 49.9%), with the actual values ranging from 19.4 μm – 32.3 μm on the abaxial and from 19.4 μm – 31.9 μm on the adaxial side. The range of SCL varies in the Sativa complex from 17.4–28.3 μm on the abaxial and from 18.6–25.4 μm on the adaxial side. SCL varies from 18.0–36.0 μm on the abaxial and from 16.1–29.7 μm on the adaxial side of leaf collectively in the species of the Meyeriana, Ridleyi and other (M-R-O) complexes.

Consistent to the stomatal length, the width (SCW) also varies almost two folds, from 11.1 μm (in *O. meridionalis*) to 21.2 μm (*O. coarctata*) on the abaxial surface and from 11.5 (*O. sativa*) to 17.7 μm (*O. australiensis*) on the adaxial surface (Table S1). Except in Meyeriana, the species of Officinalis, Ridleyi and Others (M-R- O) show greater stomatal width than the species of Sativa complex (Fig. 5). The SCW_ab_ is most diverse in the Officinalis complex (SCW_ab_ = 49.92%, SCW_ad_ = 37.5%, Table S2) and the actual value of this trait ranges from 11.6–18.8 μm on the abaxial and from 12.1–17.7 μm on the adaxial side. This is followed by species of the Sativa complex, which vary from 11.1–15.0 μm on the abaxial side, and from 11.5–13.6 μm on the adaxial side. The width varies from 11.9–21.2 μm in the abaxial and from 11.5–16.6 μm on the adaxial side collectively in the species of Meyeriana, Ridleyi and Other (M-R-O) complexes. It is easily noticeable that to compensate for the fewer number, stomata are often larger and slender in shape (in at least 70% of cases) on the adaxial surface. In *O. sativa*, the SCW_ad_ is 19% larger than SCW_ab_ (Table S1).

The diversity in SCA is even greater, as an added effect of the variation, noticed in length and width. This is the most diverse trait (GD = 147.69%) among all the traits studied, followed by SD_ad_ (91.43%) in Table S2. The species of Sativa complex contain smaller stomata in average compared to other groups (Fig. 5). SCA is significantly different in the Sativa and Officinalis complex in abaxial side, with smallest stomata in Sativa complex in average. But the SCA are similar in average in these two complexes on the adaxial side (Fig. 5). Although, both of these SCA_ad_ are quite smaller than SCA_ad_ of Ridleyi complex in the M-R-O group (Table S2). Overall, the SCA ranges from 128.0 μm^2^ (*O. meyeriana*) – 454.7 μm^2^ (*O. coarctata*) on the abaxial and from 139.1(*O. granulata*) – 323.5 μm^2^ (*O. grandiglumis*) on the adaxial side (Table S1). This trait varies from 150.1–233.9 μm^2^ on the abaxial and from 163.4–263.6 μm^2^ on the adaxial side within the Sativa complex (Table S2). This varies even greater in the species of the Officinalis complex with a range from 158.0–338.3 μm^2^ on the abaxial and from 173.4–323.5 μm^2^ on the adaxial side of the leaf. The SCA varies from 128.0–454.7 μm^2^ on the abaxial and from 139.1–322.3 μm^2^ on the adaxial side collectively in the species of the Meyeriana and Ridleyi complexes. The stomata of *Oryza coarctata* (SCA_ab_ = 454.7 μm^2^) are almost double the size of those of IR64 (SCA_ab_ = 221.6 μm^2^).

### Variation in Guard Cells (GC)

**Fig. 6:**
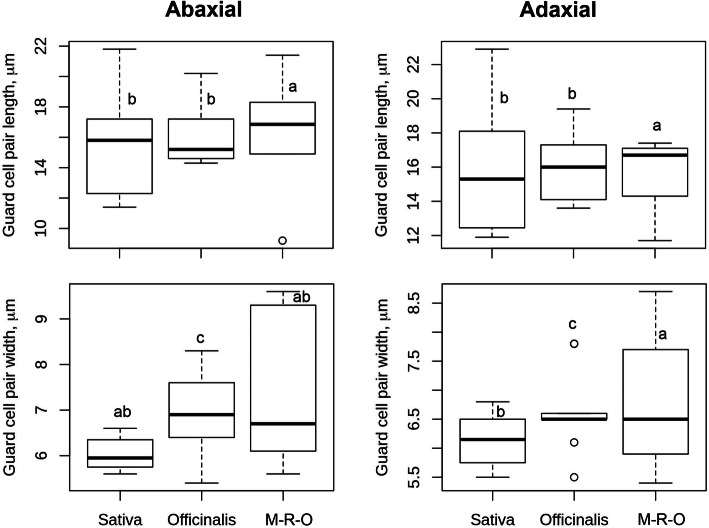
Box plots represent the diversity in guard cell size in *Oryza*; Sativa, Officinalis and M-R-O (Meyeriana, Ridleyi and Others) complexes. A detailed value scored for each species, and range of diversity for each complex is given in Table S3 and in Table S4. GCL, GCW were measured from 25 random stomatal images for each species. Boxes shows 25th to 75th percentile, horizontal line represents the median and whiskers indicate the minimum and maximum values. Different letters represent significant difference in different complexes for the trait (at *P* < 0.05). The values on the adaxial leaf side are of excluding *O. coarctata*. Sativa complex contains eight, Officinalis complex contains nine, and M-R-O contains six *Oryza* species

Guard cells, the primary determinant of the rates of gas exchange, vary significantly (*P* < 0.001) among the *Oryza* species (Fig. 6, Table S3) and *Oryza* complexes (Table S4). Distant complexes (M-R-O) show a wide range of GC pairs, where the species of Ridleyi and Other complexes contain larger GC pairs and species of Meyeriana contain smaller GC pairs (Table S4). The length of the GC pair varies from 9.1 (*O. granulata*) to 21.8 μm (*O. barthii*) on the abaxial and 11.7 (*O. granulata*) to 22.9 μm (*O. barthii*) on the adaxial leaf surface. The width of the GC pair varies from 5.4 (*O. rhizomatis*) to 9.6 (*O. brachyantha*) μm on the abaxial and from 5.4 (*O. granulata*) to 8.7 (*O. brachyantha*) μm on the adaxial leaf surface. While in most of the species the length of the guard cells are almost twice than the width, the stomata of *O. sativa*, *O. rufipogon*, *O. glaberrima*, *O. barthii* have longer guard cells than rest. In contrast, the stomata of *O. nivara*, *O. granulata*, *O. brachyantha* contain wider guard cells compared to others. Overall, the length is more diverse than the width, showing a maximum overall diversity in abaxial GCL (78.70%) and minimum diversity in adaxial GCW (50.70%) in Table S4. The GCL varies most in the Sativa complex (67.09% and 69.6% in the abaxial and adaxial side respectively), whereas, the GCW is more variable in the Officinalis complex (42.0% and 34.8% in the abaxial and adaxial side respectively).

### Variation in Stomatal Functional Traits; Maximum Conductance (*g*_max_) and Leaf Carbon Isotope Discrimination (∆^13^C)

**Fig. 7:**
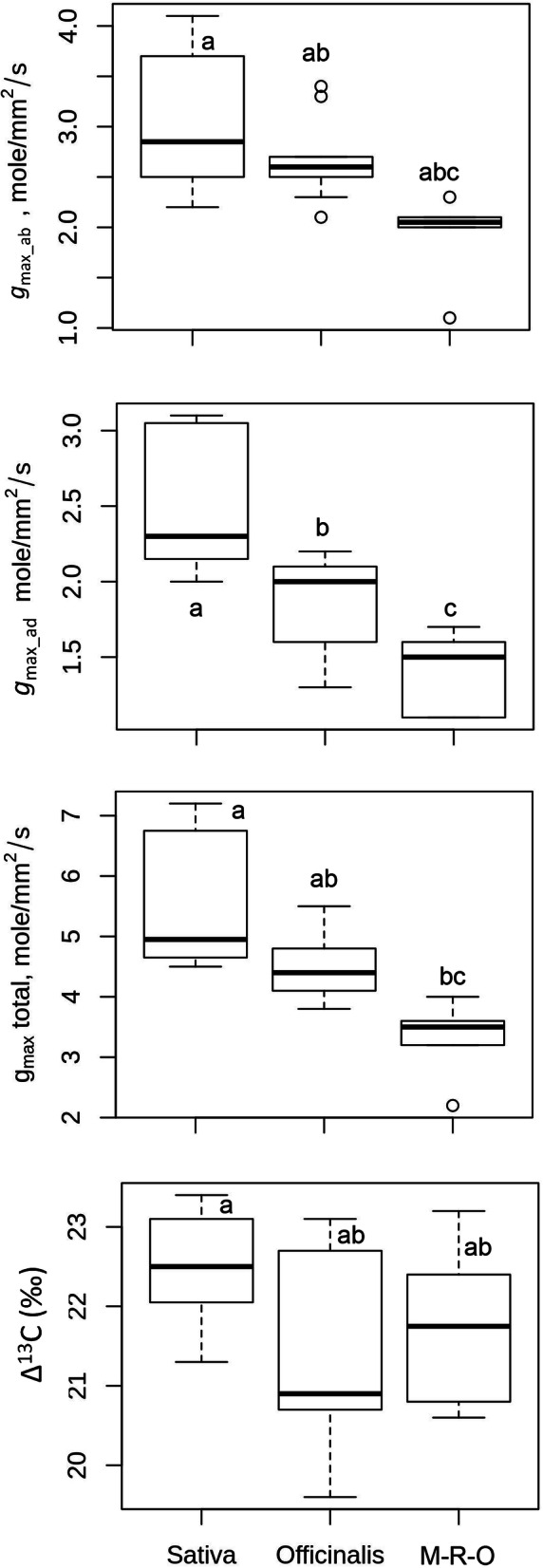
Box plots represent the diversity of stomatal maximum anatomical conductance to water vapor through abaxial and adaxial leaf surface, total conductance and carbon isotopic discrimination property of different *Oryza* complexes; Sativa, Officinalis and M-R-O (Meyeriana, Ridleyi and Others). The values of these traits for each species, and range of diversity for each complex is given in Table S5 and in Table S6. Boxes shows 25th to 75th percentile, horizontal line represents the median and whiskers indicate the minimum and maximum values. Different letters represent significant difference in *g*_max_ab_ (at *P* < 0.05), *g*_max_ad_ (at *P* < 0.001) and *g*_max_total_ (at *P* < 0.01). The values on the adaxial leaf side are of excluding *O. coarctata*. Sativa complex contains eight, Officinalis complex contains Nine, and M-R-O contains six *Oryza* species. Δ^13^C values are obtained from 3 replications for each species. Δ^13^C values differ significantly (at *P* < 0.05) among *Oryza* complexes

Significant diversity prevails in stomatal functional traits in different *Oryza* complexes (Fig. 7). Sativa complex shows the highest conductance followed by Officinalis and Other complexes. The value of ∆^13^C is also highest in Sativa complex. Overall, the maximum anatomical water conductance through stomata (*g*_max_total_) varies more than three folds among *Oryza* species (Table S5). Conductance through the abaxial side is the most diverse trait among the studied functional parameters (GD = *g*_max_ab_ 115.00%, Table S6). Except in *O. longistaminata* and *O. australiensis,* which have slightly higher conductance through the adaxial stomata*;* and in *O. granulata,* which has similar *g*_max_ values in both sides, the overall conductance through the abaxial surface (*g*_max_ab_) is 30% higher than the conductance through the adaxial surface (*g*_max_ad_), The *g*_max_ is much higher in the cultivated species than wild species, except in wild rice *O. rufipogon,* which has an intermediate value of *g*_max_total_ of 7.0 mol mm^− 2^ s^− 1^, compared to the two cultivated species *O. sativa* (*g*_max_total_ = 6.5 mol mm^− 2^ s^− 1^) and *O. glaberrima* (*g*_max_total_ = 7.2 mol mm^− 2^ s^− 1^). The African cultivated rice *Oryza glaberrima* has the highest conductance (*g*_max_total_ = 7.2 mol mm^− 2^ s^− 1^) among all the *Oryza* species. Diversity in *g*_max_total_ is highest in the Sativa complex followed by Officinalis, Meyeriana and Ridleyi complexes.

Leaf carbon isotope discrimination, (∆^13^C) values, were used as an estimate of the leaf level *WUE*_i_ (McCarroll and Loader 2004; Saurer et al. 2004). An increase in the ∆^13^C value reflects a lower *WUE*_i_ and vice versa. Many of the wild species show lower ∆^13^C or discrimination than domesticated *O. sativa* and *O. glaberrima* (Table S5). For instance, ∆^13^C is =19.6‰ in *O. grandiglumis* and is lower than the ∆^13^ C = of 23.1‰ in *O. sativa*, suggesting higher water conservation in these wild species (Table S5). Overall diversity is quite low in this trait (17%) and the lowest amongst all of the studied traits (Fig. 7, Table S6). The Officinalis complex is the most diverse (16.3%) for this trait (Table S6).

### Structure-Function Relationships in Rice Stomatal Traits

**Fig. 8:**
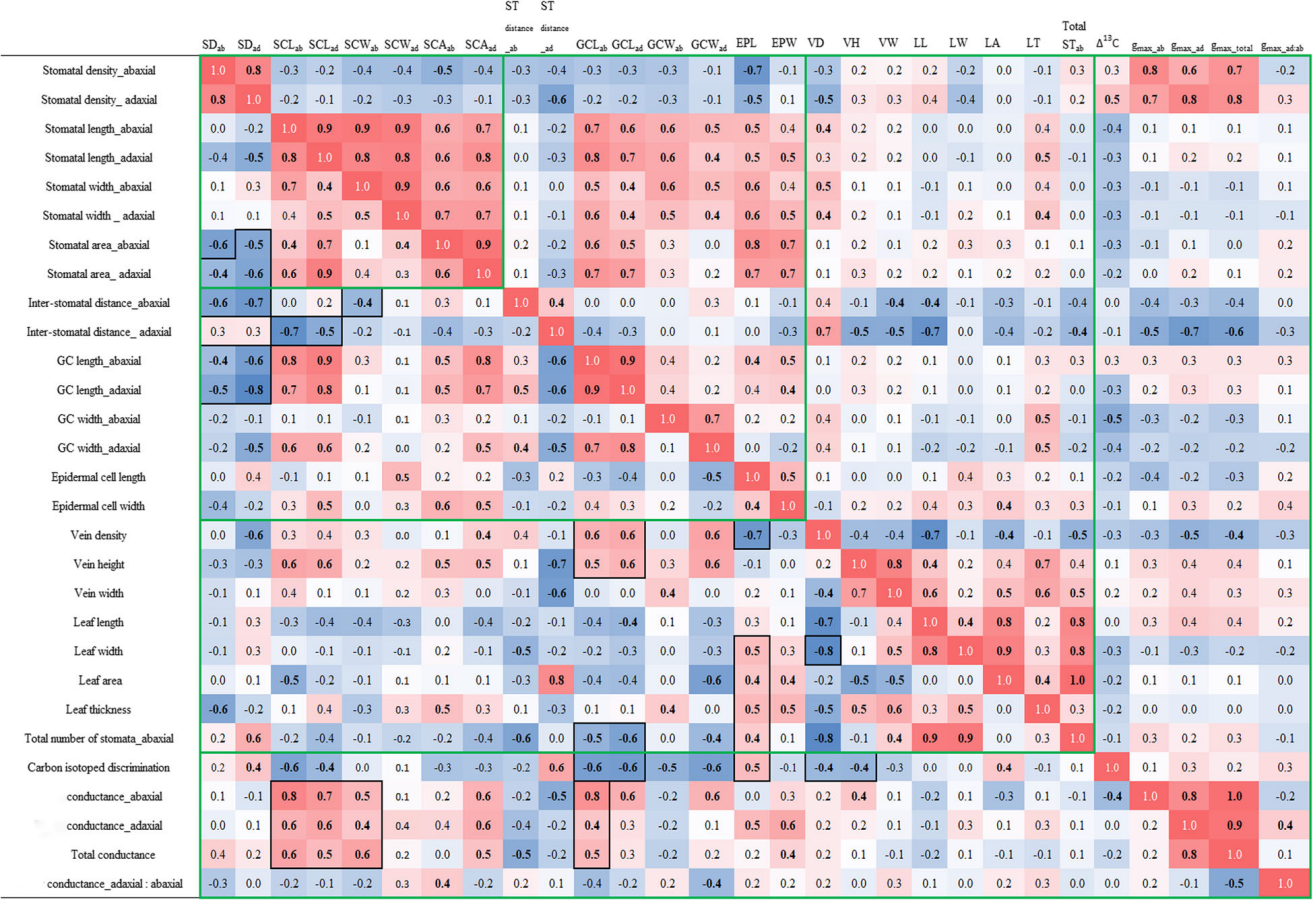
Pearson product-moment correlation values matrix of stomatal and leaf traits. Upper diagonal represents the non- corrected r values, whereas the lower diagonal represents the phylogenetically corrected r values. Cells are colour coded in shades of blue to red for negative and positive correlations respectively. Significant (*P* < 0.05) correlation r values are mentioned in “bold”. Post-correction new significant r values are bordered with black line. 1st green lined box (from the top) shows interactions among structural components of stomatal complex, 2nd green lined box shows the interactions of GC and accessory cells to SC. 3rd green lined box shows the interactions of vein and leaf related traits to SC and GC, 4th green lined box shows interactions of stomatal functional traits to SC, GC and others

In an attempt to link the stomatal structural features to its function, we have carried out a Pearson Correlation analysis (Fig. 8) followed by phylogenetic correction (as in paper of Brodribb et al. 2013). Both the normal and phylogenetically corrected “r” values are presented diagonally at the top and the bottom of the r matrix in Fig. 8, highlighting the significant correlations between traits (at *P* < 0.05). In this correlation analysis, we have included the data of the vein and leaf architecture from our previous leaf anatomy paper (Chatterjee et al. 2016) to provide a deeper understanding of how the stomatal traits vary with different leaf architectures.

**Fig. 9:**
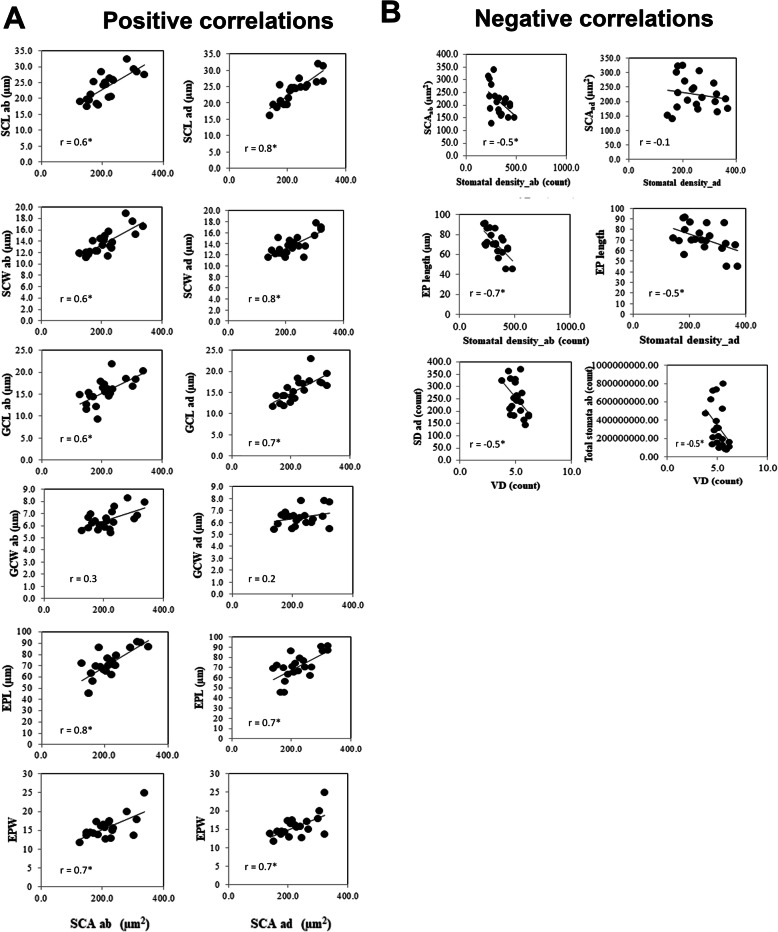
Number of positive (**a**) and negative (**b**) correlations between stomatal structural traits. Significant correlation values (r) are marked with * (at *P* < 0.05). *n* = 22

Both negative and positive associations are found to work either antagonistically or supportively to optimize the final function of a leaf. A number of important positive correlations (Fig. 8) are found within different components of the stomatal size, guard cells (GC) and epidermal cells (EP), which are all positively related to each other (Fig. 9a). Another group of positive correlations lies among the traits of vein (VW, VH) and leaf morphological components (LL, LW, LA, LT, etc.), which are positively related to each other (Fig. 8 matrix). In the upper diagonal (Fig. 8), the non- corrected r values show clear negative trends between stomatal number and all of the measured stomatal size parameters, which are statistically significant for SCA and EPL (Fig. 9b, the EP actual values are in Table S7). The inter-stomatal distance appears to be negatively related to almost all of the leaf and vein related traits (actual values of leaf traits are in Table S8, Chatterjee et al. 2016). Vein density (VD) is negatively correlated with SD, total stomata in leaf (Fig. 9b) and vein height, width and other leaf related traits, and *g*_max,_ Fig. 8 (e.g., VD: *g*_max_total_ *r* = − 0.4, *P* < 0.05).

In many instances, the r values show stronger relations after the phylogenetic correction. For example, the negative relationship of SD with SCA, GCL; the negative correlations of SC_distance_ with SD and SCL; and the negative correlations of GCL with SD, total stomata and ∆^13^C are much stronger after phylogenetic correction (Fig. 8). Similarly, some of the positive relations like: the SCL and SCA with *g*_max_; the GCL with VD, VH; and the EPL with LW, LA, LT, total stomata, ∆^13^C are stronger and very prominent after the correction (marked by thick borders in Fig. 8). The positive correlations of LT with SC and GC width suggest that thicker leaves should have bigger size stomata in rice.

**Fig. 10:**
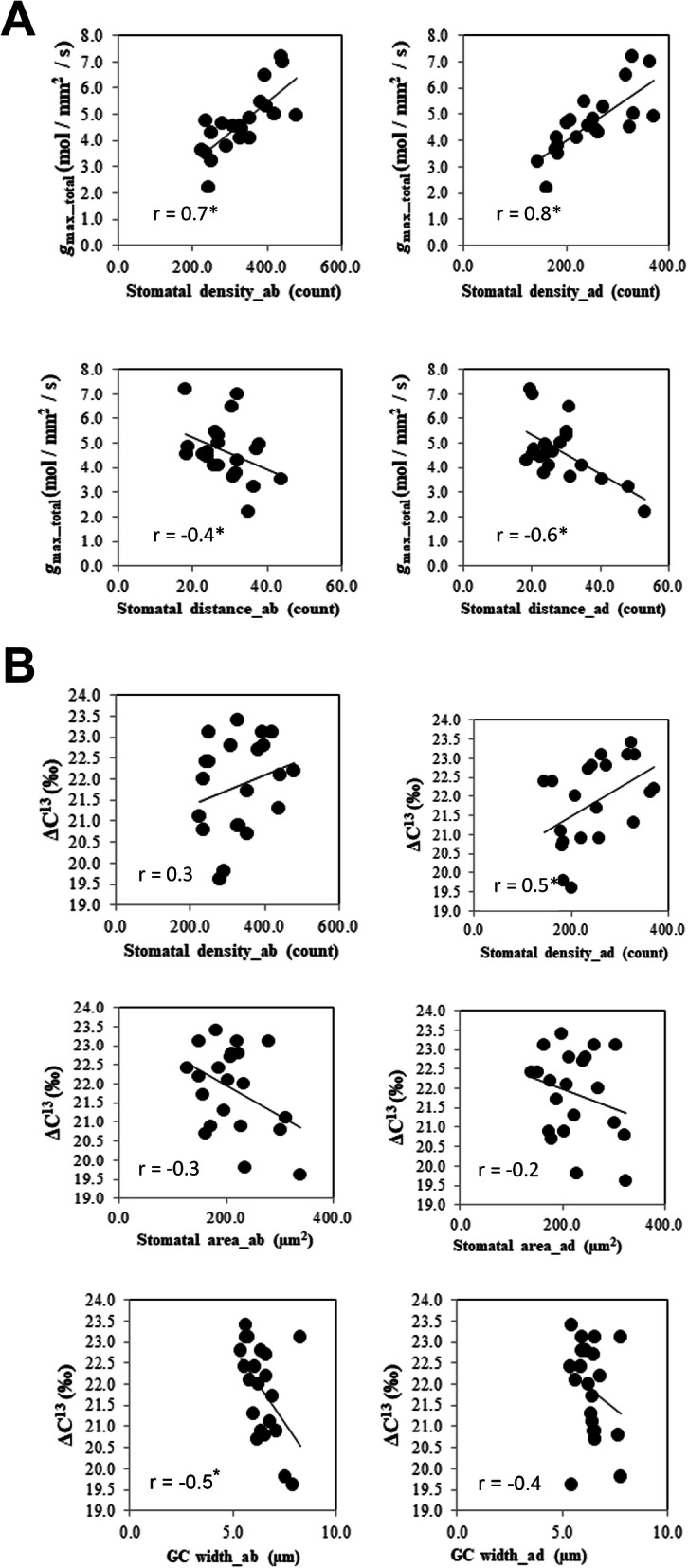
Positive interactions of stomatal density and stomatal distance with *g*_max_ (**a**), and interactions of stomatal density, stomatal area and GC width with ΔC^13^ (**b**). Significant correlation values (r) are marked with * (at *P* < 0.05). *n* = 22

Figure 8 shows the *g*_max_total_ increases with increases in both abaxial and adaxial *g*_max.._ We find the stomatal density (SD) to be positively correlated with *g*_max_ and ∆^13^C (Fig. 10a, b), which are significant for abaxial and adaxial and total *g*_max_ at *P* < 0.05. A significant negative correlation was found between SC_distance_ and *g*_max_ (example, SC_distance_: *g*_max_total_ *r* = − 0.4, *P* < 0.05, Fig. 10a). The size of the stomata and GC also increases the *g*_max_ (Fig. 8) but reduces the ∆^13^C (Fig. 10b). These correlations results clearly indicate that an increase in stomatal density, impacts in increased *g*_max_ and increased ∆^13^C values in rice.

## Discussion

Stomatal study is one of the important research areas in current rice science. Changes in stomatal features can alter the carbon and water flux of this crop and may contribute significantly to making climate resilient rice. Because stomata are the major organ controlling water and gaseous exchange in rice plants, it can be a potential target for modification in order to increase and sustain rice yield with minimal water input (Müller et al. 2018; Franks et al. 2015; Flexas 2016; Zhang et al. 2012). Being a positive regulator of photosynthesis (Kusumi et al. 2012), manipulation of stomatal traits, especially the number and size, could pave new ways to break the rice yield barrier especially in areas with limited water availability (Oshumi et al. 2007; Xu et al. 2009).

### Extensive Stomatal Diversity Is Present in *Oryza* Species

A combination of domesticated and non-domesticated rice species (Table 1) allowed us to explore stomatal diversity in a whole range of primary (close to cultivated rice), secondary (relatively distantly related) and tertiary (fairly distantly related) genetic pools of the genus *Oryza* (Vaughan et al. 2003; Sweeney and McCouch 2007). A significant amount of natural diversity in stomatal traits is documented in different *Oryza* species (Fig. 5, 6, 7) and among the different *Oryza* complexes (Tables S1, S2, S3, S4, S5, S6). Substantial diversity in the stomatal structure has been noticed in *Oryza* family from the very small stomata of *O. nivara*, *O. meridionalis, O. granulata and O. meyeriana* to the relatively very big stomata of *O. grandiglumis*, *O. ridleyi, O longiglumis* and *O. coarctata* (Fig. 2, 3, 4). Notably, genetically close species do not necessarily have a similar kind of stomatal structure, which shows that the stomatal variation in rice family does not follow a robust phylogenetic trend and might be driven by domestication or environmental factors. This is further supported by very low phylogenetic signals in the stomatal trait values (Table S9).

It is interesting to note that the evolutionary dynamics of the number and size of stomata in the *Oryza* family vary differently on different sides of the leaf and in different genetic groups or complexes. In general, there is a greater variation in stomatal number on the adaxial side but, greater variation in size on the abaxial side (Table S2). The *Oryza* species that belong to the Sativa complex or the AA genome, are more diverse in their stomatal number (SD_ab_ = 64.42%, SD_ad_ = 51.96%), whereas, the species of the Officinalis complex are more diverse in their stomatal size i.e., length, width and area (example, SCA_ab_ = 80.8%, SCA_ad_ = 65.6%). Less stomatal diversity estimated in Meyeriana and Ridleyi complexes is likely due to the limited number of members in these two complexes, although they contain unique stomatal features as evidenced from Fig. 4. It was quite expected to see the differences in the stomatal structure in the species of distant rice genomes. But interestingly, there is extensive structural diversity present even among the species of most recently evolved Sativa complex (Table S1, Table S2), where the two cultivated rice species *O. sativa* and *O. glaberrima* belong to. This clearly suggests a possibility to re-introduce favorable stomatal traits into the cultivated varieties to improve its physiology.

### Rice Has Increased *g*_max_ through an Increased Stomatal Number

Superimposing stomatal features and phylogeny of *Oryza* species, suggests no doubt that speciation has led to an increased stomatal number per unit area and a smaller stomata (Fig. S1). This is accompanied by a steady increase in both the abaxial and adaxial conductance (*g*_max_) (Fig. S2). These two observations can easily relate that the increased stomatal conductance of rice is actually due to its increasing stomatal number over time, instead of increasing the stomatal size. This is in accordance with some of the earlier reports that showed that stomata in domesticated rice are generally numerous and smaller compared to other crops **(**Chen et al. 1990; Teare et al. 1971**)** with *indica* species having more stomata than *japonica* (Maruyama and Tajima 1990).

### Structure-Function Correlations Reveal Opportunities for Adjusting the Stomatal Size to Reduce Stomatal Water Loss in Rice

Stomatal development in rice follows a typical one cell spacing pattern as in other plants (Sachs 1991; Geisler et al. 2000). Our correlation results display a negative relation between stomatal density and size (Fig. 8), associated with the reduction of the neighboring epidermal cell width. It is known that gas exchange through these stomata is highly affected by its architecture, the density and size, specially the pore depth (Fanourakis et al. 2015b), which limits the length of the diffusion pathway of gases, exchanged through these micropores (Franks and Farquhar 2001; Franks and Farquhar 2007). Our results give clear evidence that both the increased stomatal conductance and ∆^13^C in rice is caused by increasing stomatal density as these are positively related to each other. Previous reports argue that smaller stomata would respond faster and more efficiently in the uptake of CO_2_ inside the leaf, and thus will increase the photosynthesis and WUE (Lawson et al. 2014; Giday et al. 2013). But, in contrast, in our results, ∆^13^C consistently shows a significant negative relationship with stomatal and guard cell size at *P* < 0.05 (Fig. 8, Fig. 10b, Fig. S3). This suggests that the larger stomata will have less discrimination and thus theoretically will improve the *WUE*_i_ in rice. Ideally, either an increase in stomatal number or size should lead to an increase in conductance (Parkhurst 1994), which is indeed reflected by the positive relation of stomatal number and size with *g*_max_ (Fig. 8). However, considering the negative relation of GC size and ∆^13^C, we propose, increasing *g*_max_ through increased stomatal size would be a better approach to retain minimum water loss while maintaining substantial CO_2_ intake in rice, under well-watered conditions. Possibly, higher *g*_max_ through increased stomatal size would increase photosynthesis in a much better way without losing more water through transpiration. Some recent reports using genome editing also supports that reduced stomatal density actually helps in increasing the water use efficiency in rice (Caine et al. 2018). The mechanism is of course complicated and the hypothesis needs to be tested by actual field experiments. In addition, the non-significant relationship of SC area with Δ^13^C in non-corrected correlation r, suggests that there are other drivers as well controlling this physiological trait in rice. However, our result still suggests that there is scope for improving the carbon-water balance in elite rice varieties by introducing larger stomata. Several species in the Officinalis and Ridleyi complex possess larger stomata; although, it is difficult to use these species in a breeding program due to their reproductive barrier to cultivated rice. Therefore, *Oryza barthii,* that belongs to the Sativa complex, with a larger GC size of 21.8 × 6.3 μm^2^, might be the most appropriate for this purpose, as this species can be used in crossing program with more success of hybridization. Unfortunately, there is no gene directly reported for controlling stomatal size that can be edited immediately. *Epidermal Patterning Factor (EPF*) is the reported candidate gene, which controls the formation of different epidermal cells and thus also the stomata. *EPF* controls stomatal number negatively and has pleotropic effect on stomatal size. Plants overexpressed with this gene have reduced stomatal number, increased stomatal size and less adversely affected by reduced water availability (Doheny-Adams et al. 2012). Another aspect of stomatal domestication, the stomatal adaxialization (Milla et al. 2013), is not very prominent in species of Sativa complex. Although collectively the Sativa complex shows increased ad/ab conductance (Table S5), altogether the 23 species show a steady increase in total stomatal conductance through maintaining almost a consistent improvement in both abaxial and adaxial *g*_max_ in *Oryza* (Fig. S2). Hence, improving gas exchange properties through adjustment of adaxial stomatal features in rice is another viable prospect in breeding programs.

## Conclusions

Current study reports significant variation in different stomatal structural components, translated into its functional variation, which prove wild rice as a good source of diverse stomatal traits. It was interesting to find variation in the species of Sativa complex, which gives positive ideas of introgressing different stomatal features in cultivated species. We have established that rice has increased *g*_max_ by increasing SD. This is associated with reducing stomatal size and an increase in the isotopic discrimination property of this crop, an indirect measurement of the water loss by stomata. Our result indicates that larger stomata helps to maintain higher water use efficiency in rice, but the exact mechanism of water conservation in plants might be more complicated and often complemented with activities of other structural components of leaf. Moreover, the preconditioning of leaf biochemistry is also important to support altered stomatal function. As an essential documentation of wild rice stomata, we hope our result would be broadly useful in the context of the present trend to increase productivity and water use efficiency of rice crop and will kick start inclusion of stomatal traits in breeding programs.

## Material Methods

### Plant Material and Growth Condition

*Oryza* species were grown in a green house, dedicated for conducting wild rice experiments only, at the International Rice Research Institute (14.5833° N, and 120.9667° E). All species were grown from seeds (Obtained from Genetic Resource Centre, IRRI), except *O. granulata*, *O. meyeriana* and *O. coarctata*, which were grown from fresh tillers taken from mature plants. Seed dormancy was broken by placing seeds in an oven at a temperature of 45 °C for 5 days. Seeds were germinated on sterile damp filter paper in Petri-dishes in the dark at a temperature of 30 °C for 3–5 days (depends on species), followed by 2 days in the light at the same temperature. Seedlings were transplanted into the 0.5 L pots first and then 5 L pots at 29 DAS and kept in the screen house with 500 and 2000 μmol m^− 2^ s^− 1^ of irradiance (average 1000 μmol m^− 2^ s^− 1^), and a day length of between 11 and 13 h, and day and night temperature ranged between 21 and 34 °C (average 30 °C in day and 25 °C at night) with a relative humidity of between 60 and 70% (average 66%). Pots were filled with soil from the IRRI upland farm mixed with 25% volume of coco-coir and basic NPK nitrogen fertilizer. Pots were watered two times daily. Care was taken to maintain similar water and fertilizer status for all the species. For the species that were grown from the tillers; the small tillers which were comparable to the seedlings, were first transplanted to pots and maintained in the same condition as applied for the seed grown species. Each species were cultivated in 10 replications, from where three best plants were chosen for leaf sampling at the vegetative stage for stomatal study and carbon isotope analysis.

### Leaf Stomatal Imprinting for Stomatal Counting

Leaf stomatal imprints were made on either sides of the leaf (abaxial and adaxial). To avoid the spatial heterogeneity during stomatal development (Fanourakis et al. 2015a) the imprints were made exactly at the middle part of a leaf (from base to tip) and at the middle of either side of the midrib to margin. Only the youngest fully expanded leaves from the main tiller of the plants at the maximum tillering stage were sampled for all stomatal studies. Total three leaves were taken from three different plants per species. Leaf surface was first covered with clear nail varnish. When dry, the varnish layer was carefully peeled off using adhesive tape, placed on a clear glass slide and flattened by applying thumb pressure. Stomata were examined using bright-field microscopy (Olympus BX51, Olympus, Japan) and finally captured with an Olympus DP71 digital image documentation system under 20x microscopic magnification using a 10x objective (final magnification of 200x). Images were used for stomatal counting using ImageJ image processing software (Wayne Rasband, National Institute of Health, USA).

### Leaf Tissue Scraping for Stomatal Dimensions Measurements

Stomatal images, captured from the epidermal layer by leaf scraping technique, were used for stomatal complex and guard cells size and dimensions measurements. Youngest fully expanded leaves from three different plants were examined per species. Epidermal layers, taken exactly from the middle portion of any leaf in either side of the midrib were examined to record stomatal and guard cell size and dimensions. Leaf surfaces were scraped starting either from abaxial or from adaxial side of the leaf using a sharp blade in order to remove the green leaf tissues and to get clear epidermal layers of the opposite side (Sarwar and Ali 2002). These layers were mounted on a glass slide in water and then examined under 40x magnification of an Olympus BX51 microscope using a 10x objective (Final magnification 400x). Clear images were captured using an Olympus DP71 digital image documentation system attached to the microscope.

### Quantification of Stomatal Characters

Leaf stomatal traits were quantified on both the abaxial (ab) and adaxial (ad) surface of leaves. However, the deep grooves present in the adaxial surface of *O. coarctata* leaf made it extremely difficult to get clear stomatal images of that side, and therefore, was excluded from the data. Major stomatal features, including, number, length, width, area were quantified. These parameters are illustrated in Fig. 1, which shows a schematic diagram of the relative positioning of the stomatal complex (SC) on the leaf surface. The parameters are further explained in Table 2. Stomatal density (SD) was counted from 15 random images (5 images from each leaf, three leaves from each species) per species, and expressed as the number per 1 mm^2^ leaf area. Stomatal complex length (SCL, longer dimension), stomatal complex width (SCW, shorter dimension) and stomatal complex area (SCA, total area) were measured from at least 25 random stomata, taken from 3 different leaves per species. The length, width and area were measured in ImageJ image processing software according to user’s guidance. The individual stoma was selected and outlined using the option straight or segmented lines to analyze the dimension and area of the selected stoma. Guard cell length (GCL) and guard cell width (GCW) of closed GC pair were also separately measured from 25 random stomata from three different leaves per species, using ImageJ, and later used in the correlation studies and computing of stomatal maximum anatomical conductance to water. Epidermal cell length (EPL) and epidermal cell width (EPW) were measured using similar technique from 25 random images on the abaxial side of the leaf. Stomatal distance is measured as the edge to edge linear distance between two adjacent stomata along the leaf length (Fanourakis et al. 2015a). Leaf venations were observed on the adaxial surface of the leaf. The VD was counted as the number of veins in 1 mm^2^ leaf area. Veins and leaf traits were measured as described in Chatterjee et al. 2016. Traits were appropriately suffixed with -ab/−ad to refer the abaxial or adaxial nature respectively. Scoring and measurements of dimensions were performed in Image J software (Wayne Rasband, National Institute of Health, USA) according to developer’s instruction.

### Maximum Stomatal Anatomical Conductance to Water (*g*_max_)

Maximum stomatal conductance to water (*g*_max_) for each species was computed as a function of stomatal density and size (Franks and Farquhar 2001).

$$ {g}_{\mathrm{max}}=\frac{\mathrm{d}}{\mathrm{v}}\times \mathrm{SD}\times \frac{a_{\mathrm{max}}}{1+\frac{\uppi}{2}\sqrt{\frac{a_{\mathrm{max}}}{\uppi}}} $$

where, *g*_max_ is the maximum leaf stomatal conductance to water vapor (mol m^− 2^ s^− 1^); *d* is the diffusivity of water in the air (m^2^ s^− 1^); *v* is the molar volume of air (m^3^ mol^− 1^); SD is the stomatal density (stomata m^− 2^); *a* is the maximum pore area (μm^2^); and *l* is the pore depth (μm).

*l* (Pore depth) = GCW*/*2 (assuming that the stomata open in a circular shape).

and, *a*_max_ = *π*(*ρ*/2)^2^ or *a*_max_ = *π*(GCL/4)^2^ where, *ρ* is the stomatal pore length which is GCL/2 according to Franks and Farquhar 2007, Franks and Beerling 2009.

Length of the closed GC pair = GCL and the width of the closed GC pair = GCW.

### Carbon Isotope Analysis

The youngest fully expanded leaves of the main tiller from three different plants per species were collected at the maximum tillering stage for dry carbon isotope analysis. Leaves were dried in the oven for 48 h until a constant dry weight was achieved and then ground into a fine power. The dried samples (1.1–1.3 mg) was sent to Washington State University, and ^13^C/^12^C isotopic ratio (R) was determined using Micromass Isoprime isotope ratio mass spectrometer (IRMS) as described in Giuliani et al. 2013. The δ^13^C (‰) was used to represent the ^13^C/^12^C of the leaf sample relative to the isotopic reference material Vienna Pee Dee Belemnite (VPDB), and it was determined as

$$ {\updelta}^{13}\mathrm{C}\left(\raisebox{1ex}{$0$}\!\left/ \!\raisebox{-1ex}{$00$}\right.\right)=\left(\frac{{\mathrm{R}}_{\mathrm{p}}}{{\mathrm{R}}_{\mathrm{s}}}-1\right)\times 1000 $$

Where, R_p_ and R_s_ are the ^13^C/^12^C ratios of the plant leaf samples and the standard VPDB limestone, respectively. The negative value of δ^13^C was converted to a positive value of ∆ by the equation of Farquhar et al. 1989,

$$ \Delta =\frac{\left({\updelta}_{\mathrm{a}}\hbox{-} {\updelta}_{\mathrm{p}}\right)}{\left(1+{\updelta}_{\mathrm{p}}\right)} $$

Where, δ_p_ is the isotopic discrimination value of the plant and δ*a* is approximately − 8‰,

### Diversity Analysis of Stomatal Traits among the *Oryza* Species

The extent of inter-species and inter-complex genetic diversity (GD) was calculated as the range of the variation present over the average variation for each trait (Gu et al. 2014):

$$ \mathrm{GD}=\frac{\left({\mathrm{x}}_{\mathrm{max}}-{\mathrm{x}}_{\mathrm{min}}\right)}{\left(\overline{\mathrm{x}}\right)}\times 100\left(\%\right) $$

Where, *X*_max_, *X*_min_ and $\overline{X}$ stands for maximum, minimum and the mean value for the trait.

### Traits Correlation

Pair-wise correlations (*r*) between traits were performed using statistical program R (www.Rproject.org) and were plotted in a matrix. The *r* values greater than 0.43, was considered as significant at *P* < 0.05. We also considered the hidden possibility of inborn correlations between traits that may result from the close phylogenetic relationship of *Oryza* species. Therefore, phylogenetic correction was applied to the correlation values, using the method as applied in a paper of Brodribb et al. 2013, using the “pic” function (phylogenetically independent contrast) in “ape” package (Paradis et al. 2004) of R (www.Rproject.org). The correlation plots were created in Excel.

### Statistical Analysis to Detect Significant Variation in the Stomatal Traits

One way Analysis of Variance (ANOVA) was performed in STAR (IRRI’s statistical software for biometric analysis), followed by Tukey’s pairwise comparison. All graphs were drawn in Excel (Microsoft Corp., USA). Box plots are made using R program.

## Supplementary information

**Additional file 1: Figure S1.** Trend in the stomatal structural adjustment in *Oryza* family. **Figure S2.** Evolutionary trend in abaxial, adaxial and total *g*_max_, and the fraction of the –ad/−ab conductance. **Figure S3.** Correlation of Δ^13^C with stomatal and leaf traits.

**Additional file 2: Table S1.** Stomatal number, length, width and area in *Oryza* family. **Table S2.** Stomatal structural diversity in different *Oryza* complexes. **Table S3.** Diversity in guard cell length (GCL) and guard cell width (GCW) in rice family. **Table S4.** Guard cell diversity (%) in different *Oryza* complexes. **Table S5.** Abaxial and adaxial stomatal conductance (*g*_max_), and carbon isotope discrimination (Δ^13^C) values in rice family. **Table S6.** Diversity in stomatal function in different *Oryza* complexes. **Table S7.** Accessory traits: Inter-stomatal distance, epidermal cell length (EPL) and width (EPW). **Table S8.** Leaf morphological traits, vein characters and total stomata. **Table S9.** Phylogenetic signal in stomatal traits.

## Data Availability

The data sets supporting this article are included in the article and in the additional files.
