# Supplementary material for: Natural Diversity in Stomatal Features of Cultivated and Wild Oryza Species
Source: Rice (N Y). 2020 Aug 20;13:58. doi: 10.1186/s12284-020-00417-0 (PMC7441136; doi:10.1186/s12284-020-00417-0)
Supplement: Supplementary file 1 — Additional file 1: Figure S1. Trend in the stomatal structural adjustment in Oryza family. Figure S2. Evolutionary trend in abaxial, adaxial and total gmax, and the fraction of the –ad/−ab conductance. Figure S3. Correlation of Δ13C with stomatal and leaf traits. [file 12284_2020_417_MOESM1_ESM.pdf]

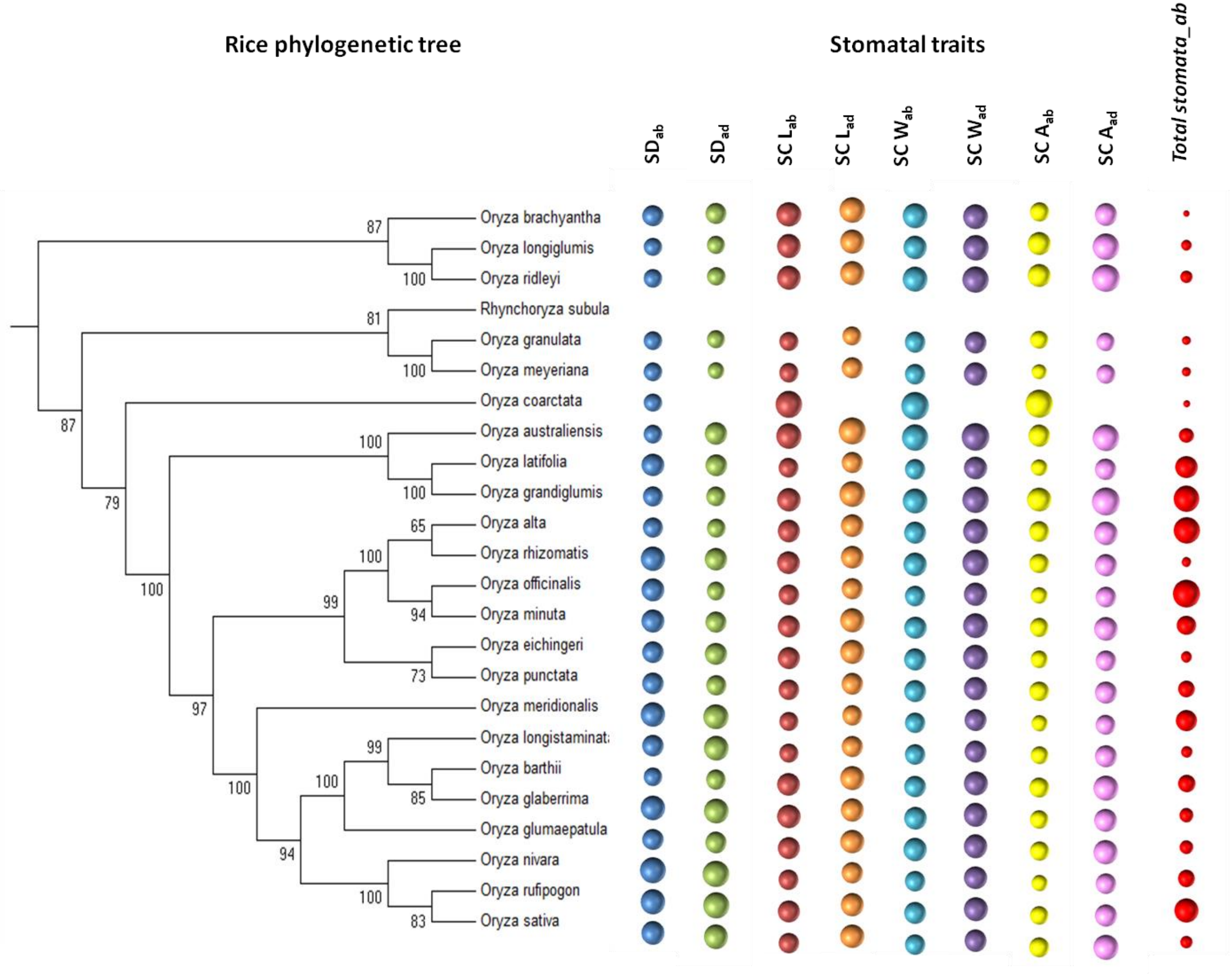

Figure S1. Trend in the stomatal structural adjustment in *Oryza* family. Left is the phylogenetic tree of the rice family. *Rhynchoryza subulata* is taken as an out group. The species are arranged from the top to bottom as evolved during rice speciation. Adjacent to the tree is the major structural traits of their stomata. Bubble sizes are proportionate to the relative values of the traits. This comparison suggests an increasing trend in the stomatal number, whereas declining trend in the stomatal size during rice speciation. Total number of stomata in a leaf (Total $Stomata_{\_ab} = SD_{ab}$ x LL x LW) shows interesting pattern, which suggests an overall increment in the stomatal number in recently evolved rice species.

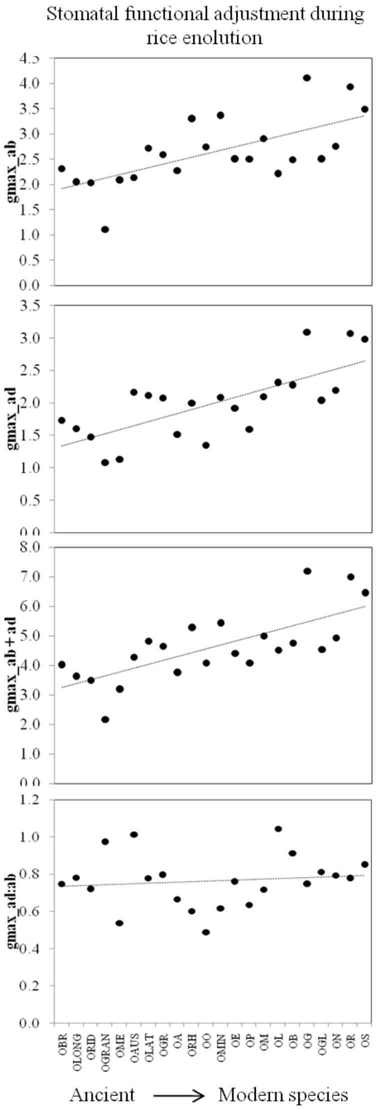

Figure S2. Evolutionary trend in abaxial, adaxial and total g_{max} , and the fraction of the –ad/-ab conductance. The *Oryza* species are arranged from most ancient to most recent (from left to right) as they appear in the phylogenetic tree (Fig. S1).

OBR= *O. brachyantha*, **OLONG** = *O. longiglumis*, **ORID** = *O. ridleyi*, **OGRAN** = *O. granulata*, **OME** = *O. meyeriana*, **OAUS**= *O. australiensis*, **OLAT** = *O. latifolia*, **OGR** = *O. grandiglumis*, **OA** = *O. alta*, **ORH**= *O. rhizomatis*, **OO**= *O. officinalis*, **OMIN**= *O. minuta*, **OE** = *O. eichingeri*, **OP** = *O. punctata*, **OM**= *O. meridionalis*, **OL**= *O. longistaminata*, **OB** = *O. barthii*, **OG** = *O. glaberrima*, **OGL** = *O. glumaepatula*, **ON** = *O. nivara*, **OR**= *O. rufipogon*, **OS** = *O. sativa*

Oryza coarctata is exempted from this analysis as only g_{max_ab} is available in our data.

	Carbon isotope discrimination	
Traits	Non-adjusted	Phylogenetically adjusted
SD_{ab}	0.3	0.2
SD_{ad}	**0.5**	**0.4**
SCL_{ab}	-0.4	**-0.6**
SCL_{ad}	-0.3	**-0.4**
SCW_{ab}	-0.3	0.0
SCW_{ad}	-0.3	0.1
SCA_{ab}	-0.3	-0.3
SCA_{ad}	-0.2	-0.3
ST $Distance_{ab}$	0.0	-0.2
ST $Distance_{ad}$	-0.1	**0.6**
GCL_{ab}	-0.4	**-0.6**
GCL_{ad}	-0.3	**-0.6**
GCW_{ab}	**-0.5**	**-0.5**
GCW_{ad}	-0.4	**-0.6**
EPL	-0.2	**0.5**
EPW	-0.1	-0.1
VD	-0.3	**-0.4**
VH	0.1	**-0.4**
VW	0.2	-0.3
LL	0.0	0.0
LW	-0.3	0.0
LA_{total}	-0.2	**0.4**
LT	-0.2	-0.1
Total ST_{ab}	-0.1	0.1
g_{max_ab}	0.1	**-0.4**
g_{max_ad}	0.3	0.0
g_{max_total}	0.2	-0.2
$g_{max\_ad:ab}$	0.3	0.0

Fig. S3. Correlation of $\Delta^{13}C$ with stomatal and leaf traits. Number in bold shows significant (*P*<0.05) correlation. Thick lined boxes show similar kind of significant association (positive / negative) between traits in both adjusted and non-adjusted correlations.
Traits codes are mentioned in Table 2.
