# Supplementary material for: Natural Diversity in Stomatal Features of Cultivated and Wild Oryza Species
Source: Rice (N Y). 2020 Aug 20;13:58. doi: 10.1186/s12284-020-00417-0 (PMC7441136; doi:10.1186/s12284-020-00417-0)
Supplement: Supplementary file 2 — Additional file 2: Table S1. Stomatal number, length, width and area in Oryza family. Table S2. Stomatal structural diversity in different Oryza complexes. Table S3. Diversity in guard cell length (GCL) and guard cell width (GCW) in rice family. Table S4. Guard cell diversity (%) in different Oryza complexes. Table S5. Abaxial and adaxial stomatal conductance (gmax), and carbon isotope discrimination (Δ13C) values in rice family. Table S6. Diversity in stomatal function in different Oryza complexes. Table S7. Accessory traits: Inter-stomatal distance, epidermal cell length (EPL) and width (EPW). Table S8. Leaf morphological traits, vein characters and total stomata. Table S9. Phylogenetic signal in stomatal traits. [file 12284_2020_417_MOESM2_ESM.pdf]

Table S1: Stomatal number, length, width and area in rice family.

Oryza Species	Stomatal density, SD / mm^2 (count)						Stomatal length, SCL (µm)						Stomatal width, SCW (µm)						Stomatal area, SCA (µm^2)					
	Abaxial ***			Adaxial ***			Abaxial ***			Adaxial ***			Abaxial ***			Adaxial ***			Abaxial ***			Adaxial ***		
	AV	SE		AV	SE		AV	SE		AV	SE		AV	SE		AV	SE		AV	SE		AV	SE	
O. sativa	393.7	± 15.16	cd	317.5	± 21.45	ab	20.2	± 0.09	hi	24.9	± 0.35	ef	11.3	± 0.11	lm	11.5	± 0.19	i	221.6	± 6.73	efg	263.6	± 7.50	b
O. rufipogon	440.5	± 12.68	ab	362.7	± 11.44	a	24.2	± 0.08	g	23.8	± 0.23	f	13.3	± 0.26	hijk	13.6	± 0.28	efg	203.7	± 4.68	efgh	208.4	± 3.80	de
O. nivara	479.8	± 8.03	a	370.2	± 10.91	bc	19.8	± 0.06	hij	20.6	± 0.28	gh	12.0	± 0.19	klm	12.3	± 0.29	ghi	150.7	± 3.56	jk	175.9	± 3.12	fgh
O. glumaepatula	307.9	± 17.46	def	243.7	± 6.68	cde	24.3	± 0.06	g	24.8	± 0.21	ef	15.0	± 0.20	efg	13.5	± 0.22	efg	211.5	± 6.28	efg	246.5	± 5.25	bc
O. glaberrima	436.5	± 9.73	abc	328.6	± 11.95	bc	28.3	± 0.08	cd	24.6	± 0.26	ef	14.5	± 0.24	efgh	13.2	± 0.26	fgh	196.9	± 5.25	fgh	224.1	± 6.56	cd
O. barthii	234.9	± 2.83	hij	208.7	± 8.64	defg	25.9	± 0.12	ef	25.4	± 0.57	def	12.7	± 0.19	jk	13.5	± 0.18	efg	233.9	± 5.09	ef	269.3	± 6.35	b
O. longistaminata	327.0	± 19.71	cd	323.8	± 21.55	ab	18.4	± 0.06	jk	19.5	± 0.18	h	12.2	± 0.14	klm	11.5	± 0.21	i	181.8	± 5.86	hij	197.8	± 5.92	def
O. meridionalis	420.0	± 12.87	bcd	331.4	± 7.10	ab	17.4	± 0.04	k	18.6	± 0.28	gh	11.1	± 0.20	lm	12.1	± 0.18	hi	150.1	± 5.90	jk	163.4	± 6.42	ghi
O. punctata	329.4	± 19.57	cde	220.6	± 5.57	defg	20.7	± 0.08	hi	21.4	± 0.21	g	13.2	± 0.22	ijk	12.4	± 0.16	ghi	228.1	± 7.29	ef	203.5	± 3.91	def
O. eichingeri	331.7	± 5.39	efg	258.7	± 10.67	defgh	25.3	± 0.10	fg	25.5	± 0.19	def	14.1	± 0.20	ghi	15.0	± 0.23	cd	172.1	± 3.53	hij	173.4	± 1.15	ghi
O. minuta	383.9	± 9.85	abc	236.6	± 6.86	def	25.1	± 0.09	fg	27.5	± 0.27	c	14.3	± 0.24	fghi	15.1	± 0.24	cd	208.6	± 4.93	efg	241.7	± 6.77	bc
O. officinalis	354.8	± 7.60	def	181.7	± 8.51	fghi	21.1	± 0.07	h	20.0	± 0.46	gh	12.2	± 0.22	kl	12.9	± 0.33	fgh	162.9	± 3.46	ij	179.3	± 5.93	fgh
O. rhizomatis	398.4	± 17.10	cde	273.0	± 9.92	cd	26.3	± 0.11	ef	24.5	± 0.27	ef	15.7	± 0.19	de	14.6	± 0.22	bc	224.0	± 4.86	efg	212.9	± 4.48	de
O. alta	289.7	± 14.11	efghi	184.1	± 4.50	efghi	25.6	± 0.08	efg	24.4	± 0.32	ef	13.8	± 0.16	ghij	14.2	± 0.10	def	236.0	± 5.06	e	228.5	± 5.52	cd
O. grandiglumis	278.6	± 9.26	ghi	201.3	± 5.52	fghi	27.4	± 0.16	de	31.2	± 0.55	ab	16.5	± 0.34	cd	17.0	± 0.41	a	338.3	± 11.47	b	323.5	± 10.12	cd
O. latifolia	351.6	± 8.64	bcd	253.2	± 15.04	def	19.4	± 0.05	hij	19.4	± 0.21	h	11.6	± 0.10	m	12.1	± 0.16	hi	158.0	± 3.87	jk	188.6	± 3.93	efg
O. australiensis	250.8	± 15.52	def	263.5	± 5.72	def	32.3	± 0.10	b	31.9	± 0.57	ab	18.8	± 0.38	b	17.7	± 0.34	a	280.0	± 10.47	d	305.6	± 6.81	a
O. meyeriana	250.8	± 5.94	ghi	143.7	± 4.83	ghi	19.1	± 0.11	ij	19.4	± 0.45	gh	11.9	± 0.31	klm	12.7	± 0.44	ghi	128.0	± 3.84	k	151.9	± 3.62	hi
O. granulata	241.3	± 3.42	ij	162.7	± 7.25	i	18.0	± 0.10	jk	16.1	± 0.22	i	12.3	± 0.25	kl	11.5	± 0.20	i	186.6	± 8.20	ghi	139.1	± 3.91	i
O. ridleyi	236.5	± 12.92	ij	184.1	± 8.18	hi	29.2	± 0.07	cd	26.5	± 0.33	cd	17.5	± 0.25	bc	16.6	± 0.39	ab	302.6	± 5.77	cd	322.3	± 9.68	a
O. longiglumis	224.3	± 10.07	ij	178.6	± 10.59	fghi	28.5	± 0.10	cd	26.4	± 0.66	cde	15.2	± 0.32	def	15.4	± 0.24	bcd	312.9	± 7.35	bc	301.0	± 6.78	a
O. brachyantha	316.7	± 1.90	efgh	223.8	± 3.01	defg	30.0	± 0.11	c	29.7	± 0.74	b	17.3	± 0.39	cd	14.6	± 0.04	cde	144.9	± 7.79	jk	181.4	± 6.04	fg
O. coarctata	227.8	± 10.85	j	-	-		36.0	± 0.13	a	-	-		21.2	± 0.56	a	-	-		454.7	± 11.31	a	-	-	

Values are presented as the average (AV) ± SE. Stomatal density (SD) were counted from total 15 images / species. Stomatal length (SL), width (SW) and area (SA) are measured from total 25 random stomata / species. *** denotes significant difference for that trait among the species at $P<0.001$. Different letters show significant difference ($P<0.05$).

Table S2: Stomatal structural diversity in different *Oryza* complexes. All traits varies significantly (***, P<0.001) among *Oryza* complexes and thus are marked with different letters.

		Stomatal density (SD)		Stomatal length (SCL)		Stomatal width (SCW)		Stomatal area (SCA)	
		Abaxial ***	Adaxial ***	Abaxial ***	Adaxial ***	Abaxial ***	Adaxial ***	Abaxial ***	Adaxial ***
Genetic diversity for the trait (%)		78.28	91.43	75.84	66.38	70.92	44.87	147.69	82.92
Sativa complex	Diversity (%)	64.42 ^{a}	51.96 ^{a}	48.78 ^{d}	29.80^{c}	30.41^{d}	16.6^{c}	43.2^{d}	46.08^{b}
	Min	234.92	208.73	17.44	18.65	11.10	11.50	150.15	163.39
	Max	479.76	370.24	28.33	25.44	14.98	13.60	233.91	263.57
	Average	380.03	310.82	22.33	22.78	12.76	12.70	193.79	217.36
	SE	29.17	19.87	1.38	0.97	0.50	0.30	10.95	13.28
Officinalis complex	Diversity (%)	44.75 ^{b}	39.62 ^{b}	51.70 ^{c}	49.90^{b}	49.92^{c}	37.5^{b}	80.8^{c}	65.68^{b}
	Min	250.79	181.75	19.44	19.43	11.62	12.10	250.79	173.37
	Max	398.41	273.02	32.26	31.95	18.84	17.70	158.04	323.49
	Average	329.87	230.31	24.79	25.08	14.46	14.90	338.30	228.54
	SE	16.34	11.62	1.32	1.50	0.75	0.70	223.11	17.90
								19.50	
Meyeriana complex	Diversity (%)	3.87 ^{C}	12.43 ^{d}	5.90 ^{e}	18.92^{d}	3.14^{e}	9.9^{d}	37.23^{e}	8.77^{c}
	Min	241.27	143.65	17.96	16.07	11.88	11.50	128.01	139.12
	Max	250.79	162.70	19.05	19.43	12.26	12.70	312.91	151.88
	Average	246.03	153.17	18.51	17.75	12.07	12.10	232.53	145.50
	SE	4.76	9.52	0.55	1.68	0.19	0.60	45.10	6.38
Ridleyi complex	Diversity (%)	5.30 ^{d}	3.06 ^{c}	2.63 ^{b}	0.38^{a}	13.80^{b}	7.5^{a}	3.33^{a}	6.84^{a}
	Min	224.29	178.57	28.46	26.44	15.24	15.40	144.86	300.96
	Max	236.51	184.13	29.22	26.54	17.50	16.60	454.74	322.29
	Average	230.40	181.35	28.84	26.49	16.37	16.00	303.79	311.63
	SE	6.11	2.78	0.38	0.05	1.13	0.60	63.33	10.66
Others	Diversity (%)	32.65 ^{e}	-	18.05 ^{a}	-	20.48^{a}	-	72.0^{b}	-
	Min	227.78	-	30.03	-	17.26	-	144.86	-
	Max	316.67	-	35.99	-	21.20	-	454.74	-
	Average	272.22	-	33.01	-	19.23	-	299.80	-
	SE	44.44	-	2.98	-	1.97	-	154.94	-

Table S3. Diversity in guard cell length (GCL) and guard cell width (GCW) in rice family.

Species	Closed GC pair length (µm)		Closed GC pair width (µm)	
	Abaxial ***	Adaxial***	Abaxial***	Adaxial***
O. sativa	16.1 ± 0.3 [ghij]	17.8 ± 0.2 [cd]	5.7 ± 0.1 [jk]	6.0 ± 0.1 [defg]
O. rufipogon	16.5 ± 0.4 [ghij]	15.2 ± 0.4 [ef]	5.9 ± 0.1 [hijk]	5.6 ± 0.1 [g]
O. nivara	11.4 ± 0.2 [l]	11.9 ± 0.2 [jk]	6.6 ± 0.1 [defg]	6.8 ± 0.1 [c]
O. glumaepatula	15.5 ± 0.3 [ij]	15.4 ± 0.5 [ef]	6.4 ± 0.1 [fghijk]	5.9 ± 0.1 [efg]
O. glaberrima	17.9 ± 0.2 [def]	18.4 ± 0.4 [bc]	6.0 ± 0.1 [ghijk]	6.4 ± 0.1 [cd]
O. barthii	21.8 ± 0.6 [a]	22.9 ± 0.6 [a]	6.3 ± 0.1 [efghijk]	6.3 ± 0.2 [cdefg]
O. longistaminata	12.1 ± 0.4 [l]	12.6 ± 0.3 [hijk]	5.6 ± 0.1 [ijk]	5.5 ± 0.1 [g]
O. meridionalis	12.5 ± 0.5 [kl]	12.3 ± 0.2 [ijk]	5.8 ± 0.1 [jk]	6.6 ± 0.1 [cde]
O. punctata	15.2 ± 0.3 [hij]	13.9 ± 0.2 [ghij]	7.1 ± 0.2 [cd]	6.6 ± 0.1 [cdef]
O. eichingeri	14.3 ± 0.3 [jk]	14.1 ± 0.3 [fgh]	6.4 ± 0.1 [defghi]	6.5 ± 0.1 [cdef]
O. minuta	17.2 ± 0.5 [defg]	17.1 ± 0.2 [cde]	6.6 ± 0.1 [defgh]	6.5 ± 0.1 [cde]
O. officinalis	14.5 ± 0.3 [jk]	14.2 ± 0.4 [fg]	6.2 ± 0.1 [efghij]	6.6 ± 0.1 [cde]
O. rhizomatis	14.6 ± 0.3 [ij]	13.6 ± 0.1 [ghi]	5.4 ± 0.3 [fghijk]	6.1 ± 0.1 [cdefg]
O. alta	16.2 ± 0.3 [fghi]	17.3 ± 0.3 [cde]	7.6 ± 0.2 [bc]	7.8 ± 0.3 [a]
O. grandiglumis	20.2 ± 0.3 [bc]	19.4 ± 0.4 [b]	7.9 ± 0.1 [bc]	5.5 ± 0.4 [g]
O. latifolia	15.2 ± 0.4 [ghij]	16.0 ± 0.6 [de]	6.9 ± 0.1 [cd]	6.5 ± 0.2 [bc]
O. australiensis	18.5 ± 0.4 [cd]	17.3 ± 0.5 [cde]	8.3 ± 0.2 [b]	7.8 ± 0.2 [a]
O. meyeriana	14.9 ± 0.3 [ij]	14.3 ± 0.3 [fg]	5.6 ± 0.1 [k]	5.9 ± 0.2 [fg]
O. granulata	9.2 ± 0.3 [l]	11.7 ± 0.3 [k]	6.1 ± 0.1 [fghijk]	5.4 ± 0.2 [g]
O. ridleyi	16.7 ± 0.2 [efgh]	16.7 ± 0.4 [cde]	6.6 ± 0.2 [def]	7.7 ± 0.2 [ab]
O. longiglumis	18.3 ± 0.2 [de]	17.4 ± 0.3 [cd]	6.8 ± 0.2 [de]	6.5 ± 0.1 [cdef]
O. brachyantha	17.0 ± 0.4 [defg]	17.1 ± 0.4 [cde]	9.6 ± 0.3 [a]	8.7 ± 0.0 [a]
O. coarctata	21.4 ± 0.4 [ab]	- - -	9.3 ± 0.2 [a]	- - -

Values are presented as average ± SE of 25 random observations.

*** denotes significant difference for that trait among the species at $P<0.001$. Different letters show significant difference at $P<0.05$.

Table S4: Guard cell diversity (%) in different *Oryza* complexes.

		GC length		GC width	
		Abaxial ***	Adaxial ***	Abaxial ***	Adaxial ***
	Overall genetic diversity (%)	78.70	70.80	62.60	50.70
Sativa complex	Diversity (%)	67.09 [c]	69.6 [b]	16.6 [c]	21.3 [d]
	Min	11.40	11.90	5.60	5.50
	Max	21.80	22.90	6.60	6.80
	Average	15.50	15.80	6.00	6.10
	SE	0.06	0.07	0.01	0.01
Officinalis complex	Diversity (%)	36.4 [c]	36.4 [b]	42 [b]	34.8 [c]
	Min	14.30	13.60	5.40	5.50
	Max	20.20	19.40	8.30	7.80
	Average	16.20	15.90	6.90	6.60
	SE	0.04	0.04	0.01	0.01
Meyeriana complex	Diversity (%)	47.5 [d]	20.0 c	8.6 [c]	8.92 [e]
	Min	9.20	11.70	5.60	5.40
	Max	14.90	14.30	6.10	5.90
	Average	12.00	13.00	5.80	5.60
	SE	0.07	0.03	0.03	0.01
Ridleyi complex	Diversity (%)	9.1 [b]	4.1 [a]	2.9 [b]	16.9 [b]
	Min	16.70	16.70	6.60	6.50
	Max	18.30	17.40	6.80	7.70
	Average	17.50	17.00	6.70	7.10
	SE	0.02	0.01	0.01	0.01
Others	Diversity (%)	22.9 [a]	-	18.05 [a]	-
	Min	17.00	-	9.30	-
	Max	21.40	-	9.60	-
	Average	19.20	-	9.50	-
	SE	0.06	-	0.00	-

*** denotes significant difference for that trait among the species at $P<0.001$. Different letters show significant difference ($P<0.05$)

Table S5: Abaxial and adaxial stomatal conductance (g_{max}), and carbon isotope discrimination ($\Delta^{13}C$) values in rice family.

Species	g_{max} (mol/ mm^2/s)				Carbon isotope ratio‰ ($\Delta^{13}C$) ***			
	Abaxial	Adaxial	Total (Ab+Ad)	Ratio (Ad:Ab)				
O. sativa	3.5	3.0	6.5	0.9	23.1	±	0.6	c
O. rufipogon	3.9	3.1	7.0	0.8	22.1	±	2.0	abc
O. nivara	2.8	2.2	4.9	0.8	22.2	±	0.7	abc
O. glumaepatula	2.5	2.0	4.5	0.8	22.8	±	0.9	bc
O. glaberrima	4.1	3.1	7.2	0.8	21.3	±	0.5	abc
O. barthii	2.5	2.3	4.8	0.9	22.0	±	0.4	abc
O. longistaminata	2.2	2.3	4.5	1.0	23.4	±	0.8	c
O. meridionalis	2.9	2.1	5.0	0.7	23.1	±	0.2	bc
O. punctata	2.5	1.6	4.1	0.6	20.9	±	1.0	abc
O. eichingeri	2.5	1.9	4.4	0.8	20.9	±	0.6	abc
O. minuta	3.4	2.1	5.5	0.6	22.7	±	0.4	abc
O. officinalis	2.7	1.3	4.1	0.5	20.7	±	0.5	abc
O. rhizomatis	3.3	2.0	5.3	0.6	22.8	±	1.5	bc
O. alta	2.3	1.5	3.8	0.7	19.8	±	1.7	ab
O. grandiglumis	2.6	2.1	4.7	0.8	19.6	±	1.2	a
O. latifolia	2.7	2.1	4.8	0.8	21.7	±	0.6	abc
O. australiensis	2.1	2.2	4.3	1.0	23.1	±	0.3	bc
O. meyeriana	2.1	1.1	3.2	0.5	22.4	±	0.3	abc
O. granulata	1.1	1.1	2.2	1.0	22.4	±	0.3	abc
O. ridleyi	2.0	1.5	3.5	0.7	20.8	±	0.6	abc
O. longiglumis	2.0	1.6	3.6	0.8	21.1	±	0.7	abc
O. brachyantha	2.3	1.7	4.0	0.7	20.6	±	1.1	abc
O. coarctata	2.1	-	-	-	23.2	±	0.6	c

Stomatal conductance (g_{max}) was calculated separately for abaxial and adaxial side of the leaf. Total stomatal conductance (g_{max_Total}) to water was calculated by adding the abaxial and adaxial values. Carbon isotope discrimination values are presented as the average of 3 replications ± SD

*** denotes significant difference for that trait among the species at $P<0.001$. Different letters show significant difference ($P<0.05$)

Table S6: Diversity in stomatal function in different *Oryza* complexes.

		g_{max} (mol/ mm^2/s)			Δ^{13}C *
		Abaxial *	Adaxial ***	Total **	
	Overall diversity (%)	115.00	100.00	100.8	17.3
Sativa complex	Diversity (%)	61.2 [a]	44.0 [a]	48.2 [a]	9.3 [b]
	Min	2.20	2.00	4.5	21.3
	Max	4.10	3.10	7.2	23.4
	Average	3.10	2.50	5.6	22.5
	SD	0.70	0.40	1.1	0.7
Officinalis complex	Diversity (%)	48.1[ab]	47.3 [b]	37.7 [ab]	16.3 [ab]
	Min	2.10	1.30	3.8	19.6
	Max	3.40	2.20	5.5	23.1
	Average	2.70	1.90	4.5	21.4
	SD	0.40	0.30	0.5	1.2
Meyeriana complex	Diversity (%)	62.5 [c]	0 b[c]	37 [c]	0 [b]
	Min	1.10	1.10	2.2	22.4
	Max	2.10	1.10	3.2	22.4
	Average	1.60	1.10	2.7	22.4
	SD	0.60	0.03	0.7	0
Ridleyi complex	Diversity (%)	0 [bc]	6.6 [c]	2.7 [bc]	1.5 [a]
	Min	2.00	1.50	3.5	20.8
	Max	2.00	1.60	3.6	21.1
	Average	2.00	1.50	3.6	20
	SD	0.01	0.09	0.1	0.2
Others	Diversity (%)	9.09 [abc]	-	-	11.8 [ab]
	Min	2.10	-	-	20.6
	Max	2.30	-	-	23.2
	Average	2.20	-	-	21.9
	SD	0.10	-	-	1.8

*, **, and *** denotes significant difference for that trait among the species at $P<0.05$, 0.01 and $P<0.001$ respectively. Different letters show significant difference ($P<0.05$)

Table S7. Accessory traits: Inter-stomatal distance, epidermal cell length (EPL) and width (EPW).

	Species	Genome	Stomata - stomata Distance (µm)		Epidermal Cell Length (µm)	Epidermal Cell Width (µm)
			Abaxial	Adaxial	Abaxial	Abaxial
Asian species	*Oryza sativa*	AA	30.6 ± 0.57	30.9 ± 0.53	62 ± 0.14	17 ± 0.74
	O. rufipogon	AA	32.1 ± 0.63	20.1 ± 0.60	65 ± 0.11	16 ± 0.62
	O. nivara	AA	37.9 ± 0.94	24.0 ± 0.64	45 ± 0.22	14 ± 0.07
	O. glumaepatula	AA	18.3 ± 0.24	20.3 ± 0.35	70 ± 0.08	13 ± 0.62
	O. glaberrima	AA	18.0 ± 0.62	19.5 ± 0.62	66 ± 0.10	16 ± 0.67
African species	*O. barthii*	AA	37.0 ± 0.53	20.7 ± 0.40	70 ± 0.10	15 ± 0.63
	O. longistaminata	AA	22.6 ± 0.75	23.9 ± 0.59	86 ± 0.11	17 ± 0.93
	O. meridionalis	AA	26.8 ± 0.57	28.4 ± 0.09	45 ± 0.21	14 ± 0.05
	O. punctata	BB	26.9 ± 0.83	24.9 ± 0.11	71 ± 0.08	13 ± 0.57
	O. eichingeri	CC	24.0 ± 0.72	22.6 ± 0.85	69 ± 0.13	14 ± 0.68
	O. minuta	BBCC	26.1 ± 0.69	30.0 ± 0.47	76 ± 0.11	16 ± 0.73
	O. officinalis	CC	25.8 ± 0.56	34.6 ± 0.25	56 ± 0.08	14 ± 0.64
	O. rhizomatis	CC	26.9 ± 0.63	30.3 ± 0.53	74 ± 0.11	17 ± 0.77
	O. alta	CCDD	31.8 ± 0.95	23.5 ± 0.30	79 ± 0.08	16 ± 0.49
	O. grandiglumis	CCDD	23.8 ± 0.80	26.2 ± 0.95	87 ± 0.16	25 ± 1.02
	O. latifolia	CCDD	18.9 ± 0.52	24.3 ± 0.99	63 ± 0.07	14 ± 0.88
	O. australiensis	EE	32.1 ± 0.34	18.5 ± 0.24	86 ± 0.11	20 ± 0.58
Meyeriana Complex	*O. meyeriana*	GG	36.3 ± 1.32	48.4 ± 1.16	72 ± 0.08	12 ± 0.91
	O. granulata	GG	35.2 ± 0.82	53.0 ± 0.72	69 ± 0.07	14 ± 0.79
Ridleyi complex	*O. ridleyi*	HHJJ	43.8 ± 0.31	40.5 ± 0.31	91 ± 0.08	14 ± 0.79
	O. longiglumis	HHJJ	30.8 ± 0.66	31.4 ± 1.24	91 ± 0.13	18 ± 1.02
Others	*O. brachyantha*	FF	29.6 ± 0.24	45.0 ± 1.83	60.5 ± 0.27	12 ± 0.04
	O. coarctata	KKLL	32.2 ± 1.13	- - -	71 ± 0.07	13 ± 0.58

Values are presented as average ± SE of 25 random observations.

TableS8. Leaf morphological traits, vein characters and total stomatal.

Groups		Species	Genome	Vein: Density	Vein: Height	Vein: Width	Leaf: Length	Leaf: Width	Leaf: Area (LL X LW)	Leaf: Thickness	Total Number of Stomates in leaf abaxial side (LL x LW x SD_{ab}, count)
				Count	µm	µm	cm	cm	cm2	µm	
Sativa complex		*Oryza sativa*	AA	5.0 ± 0.9	29.5 ± 3.3	22.1 ± 3.6	41.0 ± 8.5	1.0 ± 0.1	39.6	74.0 ± 5.7	156016931.22
	Asian species	*O. rufipogon*	AA	4.4 ± 0.5	40.31 ± 5.44	31.83 ± 4.09	74.5 ± 10.1	1.9 ± 0.2	141.5	91.8 ± 9.2	623159285.71
		O. nivara	AA	5.5 ± 0.9	29.54 ± 2.79	25.51 ± 2.2	47.6 ± 7.1	0.8 ± 0.1	39.6	67.0 ± 3.9	190127865.96
		O. glumaepatula	AA	5.1 ± 0.4	44.76 ± 6.23	40.06 ± 3.7	68.1 ± 7.8	1.5 ± 0.3	101.4	85.8 ± 4.5	312278032.53
		O. glaberrima	AA	5.0 ± 0.9	34.41 ± 4.3	31.01 ± 3.8	60.7 ± 6.5	1.2 ± 0.2	72.8	65.7 ± 7.7	317777777.78
	African species	*O. barthii*	AA	4.5 ± 0.7	33.33 ± 3.55	28.95 ± 3.2	59.8 ± 4.5	1.0 ± 0.1	58.4	72.7 ± 6.3	137309660.98
		O. longistaminata	AA	3.8 ± 0.4	27.78 ± 4.47	32.68 ± 4.6	89.4 ± 11.8	1.6 ± 0.2	145.1	84.5 ± 4.0	474449931.41
		O. meridionalis	AA	4.6 ± 0.9	39.38 ± 2.97	33.56 ± 5.3	53.2 ± 11.1	0.9 ± 0.1	50.3	93.3 ± 7.2	211114814.81
Officinalis Complex		*O. punctata*	BB	4.7 ± 0.7	20.78 ± 2.74	17.16 ± 2.4	62.2 ± 3.4	1.4 ± 0.1	87.1	53.8 ± 4.2	286811111.11
		O. eichingeri	CC	5.2 ± 0.7	24.75 ± 3.74	20.99 ± 3.1	24.6 ± 3.3	1.2 ± 0.1	28.9	78.8 ± 11.2	95944228.88
		O. minuta	BBCC	5.5 ± 0.4	37.5 ± 3.3	31.85 ± 3.9	25.6 ± 3.8	1.3 ± 0.2	33.5	109.7 ± 10.9	128640249.38
		O. officinalis	CC	4.9 ± 0.5	28.19 ± 3.01	25.01 ± 2.8	58.2 ± 9.9	1.9 ± 0.3	110.6	86.5 ± 9.8	392445502.65
		O. rhizomatis	CC	5.6 ± 0.4	29.55 ± 4.84	27.88 ± 4.3	68.7 ± 11.6	2.9 ± 0.4	199.9	82.0 ± 3.5	796412228.10
		O. alta	CCDD	4.6 ± 0.4	47.72 ± 47.72	36.92 ± 4.5	75.8 ± 7.1	3.3 ± 0.7	248.5	142.5 ± 31.1	719726053.33
		O. grandiglumis	CCDD	5.5 ± 0.6	31.75 ± 4.45	29.24 ± 3.9	72.0 ± 8.8	2.6 ± 0.6	188.0	96.3 ± 7.1	523706750.82
		O. latifolia	CCDD	4.9 ± 1.03	28.24 ± 2.83	32.72 ± 2.9	78.3 ± 4.3	2.7 ± 0.3	208.8	84.9 ± 4.4	734114285.71
		O. australiensis	EE	5.1 ± 0.6	37.5 ± 2.01	31.95 ± 2.3	62.7 ± 3.9	1.4 ± 0.1	89.1	116.9 ± 15.4	223518909.63
Meyeriana Complex		*O. meyeriana*	GG	5.9 ± 1.09	22.29 ± 1.86	17.6 ± 2.2	16.3 ± 1.5	1.9 ± 0.2	30.7	69.3 ± 5.1	76919341.56
		O. granulata	GG	5.8 ± 0.5	25 ± 1.5	23.25 ± 1.7	17.7 ± 2.1	1.9 ± 0.2	34.0	61.2 ± 3.5	81933450.91
Ridleyi complex		*O. ridleyi*	HHJJ	6.2 ± 0.7	29.5 ± 1.5	22.51 ± 2.6	36.0 ± 3.6	1.9 ± 0.2	66.8	74.9 ± 4.2	157987301.59
		O. longiglumis	HHJJ	6.2 ± 0.5	26.44 ± 3.17	25.11 ± 4.04	32.2 ± 2.9	1.5 ± 0.1	49.4	75.3 ± 3.8	110813756.61
Others		*O. brachyantha*	FF	6.7 ± 0.7	25 ± 2.33	27.78 ± 4.9	18.8 ± 1.1	0.6 ± 0.1	12.1	107.3 ± 25.7	38320576.13
		O. coarctata	KKLL	5.0 ± 0.4	42 ± 3.45	36.5 ± 4.3	30.4 ± 2.0	0.7 ± 0.1	20.6	375.8 ± 11.0	47000960.22

Values are the average of the three leaves per species ± SD. Total stomatal number was calculated multiplying leaf area (LA) by the stomatal density (SD).

Table S9: Phylogenetic signal in the traits.

TRAITS	Correlation (between "phylogenetic distance" and contrasts)	Phylosig (method="K")
Stomatal Frequency_Abaxial / mm2	0.096	0.05
Stomatal Frequency_ Adaxial / mm2	0.172	0.07
ST Aperture Length_Abaxial (µm)	0.184	0.045
Stomatal Aperture Length_Adaxial (µm)	0.199	0.06
Stomatal Aperture Width_Abaxial (µm)	0.221	0.14
Stomatal Aperture Width _ Adaxial (µm)	0.134	0.12
Stomatal Area_Abaxial (µm2)	0.225	0.09
Stomatal Area_ Adaxial (µm2)	0.208	0.08
Stomatal - stomate Distance_Abaxial (µm)	0.072	0.03
Stomate - stomate Distance_ Adaxial (µm)	0.594	0.36
Length of closed GC pair_Abaxial (µm)	-0.019	0.02
Length of total closed GC pair_Adaxial (µm)	-0.08	0.02
Width of closed GC pair_Abaxial (µm)	0.386	0.21
Width of closed GC pair_Adaxial (µm)	0.345	0.11
Epidermal Cell Length_Abaxial (µm)	0.04	0.06
Epidermal Cell Width_Abaxial (µm)	0.09	0.1
Vein Density (count)	0.511	0.17
Vein Height (µm)	0.034	0.06
Vein Width (µm)	0.139	0.1
Leaf Length (cm)	0.355	0.1
Leaf Width (cm)	0.022	0.09
Total Leaf Area (LL X LW)	-0.012	0.05
Leaf Thickness (µm)	0.04	0.09
Total Number of Stomates in leaf_Abaxial (cour	0.012	0.04
Carbon Isotope Ratio‰	0.021	0.07
gmax_Abaxial (mol/ mm2/s)	0.089	0.04
gmax_Adaxial (mol/ mm2/s)	0.216	0.11
gmax_Abaxial + Adaxial (mol/ mm2/s)	0.173	0.05
gmax_Adaxial : Abaxial (ratio)	0.087	0.18
